# STARD1 Functions in Mitochondrial Cholesterol Metabolism and Nascent HDL Formation. Gene Expression and Molecular mRNA Imaging Show Novel Splicing and a 1:1 Mitochondrial Association

**DOI:** 10.3389/fendo.2020.559674

**Published:** 2020-10-20

**Authors:** Michele Campaigne Larsen, Jinwoo Lee, Joan S. Jorgensen, Colin R. Jefcoate

**Affiliations:** ^1^Department of Cell and Regenerative Biology, University of Wisconsin School of Medicine and Public Health, Madison, WI, United States; ^2^Endocrinology and Reproductive Physiology Program, University of Wisconsin School of Medicine and Public Health, Madison, WI, United States; ^3^Department of Comparative Biosciences, University of Wisconsin School of Veterinary Medicine, Madison, WI, United States

**Keywords:** START proteins, cholesterol trafficking, SIK1, CRTC2, nascent HDL formation, sm-FISH

## Abstract

STARD1 moves cholesterol (CHOL) from the outer mitochondrial membrane (OMM) to the inner membrane (IMM) in steroidogenic cells. This activity is integrated into CHOL trafficking and synthesis homeostasis, involving uptake through SR-B1 and LDL receptors and distribution through endosomes, ER, and lipid droplets. In adrenal cells, STARD1 is imported into the mitochondrial matrix accompanied by delivery of several hundred CHOL molecules. This transfer limits CYP11A1-mediated generation of pregnenolone. CHOL transfer is coupled to translation of STARD1 mRNA at the OMM. In testis cells, slower CHOL trafficking seems to be limiting. STARD1 also functions in a slower process through ER OMM contacts. The START domain of STARD1 is utilized by a family of genes, which includes additional STARD (forms 3–6) and GRAMD1B proteins that transfer CHOL. STARD forms 2 and 7 deliver phosphatidylcholine. STARD1 and STARD7 target their respective activities to mitochondria, *via* N-terminal domains (NTD) of over 50 amino acids. The NTD is not essential for steroidogenesis but exerts tissue-selective enhancement (testis>>adrenal). Three conserved sites for cleavage by the mitochondrial processing protease (MPP) generate three forms, each potentially with specific functions, as demonstrated in STARD7. STARD1 is expressed in macrophage and cardiac repair fibroblasts. Additional functions include CHOL metabolism by CYP27A1 that directs activation of LXR and CHOL export processes. STARD1 generates 3.5- and 1.6-kb mRNA from alternative polyadenylation. The 3.5-kb form exclusively binds the PKA-induced regulator, TIS11b, which binds at conserved sites in the extended 3’UTR to control mRNA translation and turnover. STARD1 expression also exhibits a novel, slow splicing that delayed splicing delivery of mRNA to mitochondria. Stimulation of transcription by PKA is directed by suppression of SIK forms that activate a CRTC/CREB/CBP promoter complex. This process is critical to pulsatile hormonal activation *in vivo*. sm-FISH RNA imaging shows a flow of single STARD1 mRNA particles from asymmetric accumulations of primary transcripts at gene loci to 1:1 complex of 3.5-kb mRNA with peri-nuclear mitochondria. Adrenal cells are similar but distinguished from testis cells by appreciable basal expression prior to hormonal activation. This difference is conserved in culture and *in vivo*.

## Highlights

- In adrenal cells, STARD1 activates translation-coupled NTD-directed cholesterol import into mitochondria for metabolism by CYP11A1. In testis cells, translation is not rate limiting.
- Species comparisons of STARD1 mRNA show specific conservation of extended 3’UTR elements that bind TIS11b, a regulator of mRNA stability and translation.
- STARD1 shows slow splicing and mRNA processing. RNA imaging quantifies asymmetric generation of primary and spliced transcripts at individual gene loci in single cells.
- Imaging of STARD1 mRNA particles identifies single molecules moving from gene loci to form a 1:1 association with perinuclear mitochondria.
- The STARD1 NTD has three conserved MPP cleavage sites coupled with positive charge clusters. Distinct cleavage forms may contribute to alternative activities: LXR activation of trafficking and apoptosis suppression.

## Introduction

Cholesterol (CHOL) has essential, diverse signaling functions through local direct effects on membrane characteristics and through metabolism. Metabolic signaling derives from bile acids, steroid hormones and hydroxyl-CHOL (HO-CHOL) derivatives, which each activate nuclear receptors and other signaling proteins. The liver and steroidogenic organs take up CHOL from HDL through the SR-B1 receptor, but also export CHOL *via* ATP-dependent pumps, notably, ABCA1 and ABCG1. STARD1 activates CHOL transfer into mitochondria to initiate steroidogenesis, *via* CYP11A1-mediated CHOL conversion to pregnenolone (1). An alternative conversion to 27HO-CHOL by CYP27A1 (2) activates LXRα, thereby inducing genes that promote CHOL trafficking, including export (3, 4). STARD1 has long been recognized to play a central role in CHOL trafficking and exhibits a breadth of unusual regulation (5, 6).

**Figure 1:**
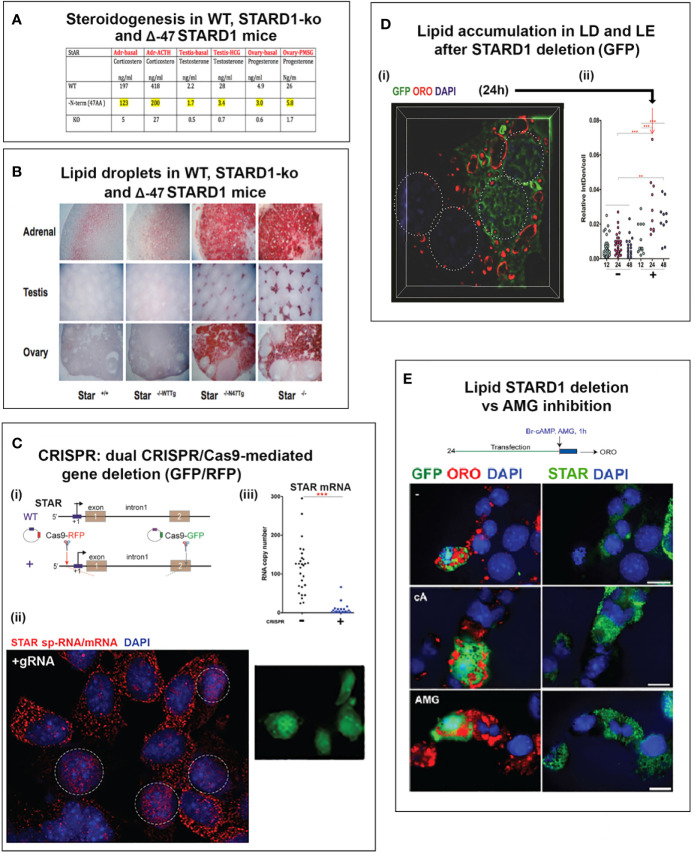
Impact of specific deletion of NTD from STARD1, in vivo, and of 1.8-kb in situ CRISPR STARD1 gene deletion in culture on cholesterol ester distribution. **(A)** Selective effects of NTD deletion on in vivo mitochondrial CHOL metabolism. **(B)** NTD deletion effects on LD accumulation match those for full STAR deletion. Sections A and B adapted from (10) **(C)** (i). Design for in situ CRISPR STARD1 deletion, which removes 1.8 kb of gene from RFP/GFP dual-marked cells. (ii and iii). After dual transfection (24 h), marked cells lose mRNA produced by 180-min of Br-cAMP stimulation. Compared to mean for adjacent unmarked cells (or g-RNA-null control cultures). **(D)** Effect after activation of 24-h CRISPR deletion of STARD1 in Y-1 cells (GFP Green) on neutral lipid accumulations ((+) gRNA; Quantified by cell fluorescence at 12, 24, and 48 h). (i). Oil red O (ORO) measured after 1-h stimulation by Br-cAMP following 24 h of CRISPR activation. Four adjacent cells: left pair, non-transfected; right pair, GFP+/transfected. (ii). Dot blot of ORO responses; Relative Integrated Optical Densities per individual cell (Int Dens/Cell). **(E)** Compare 1 h effects of Br-cAMP with deletion of STARD1 versus inhibition of CYP11A1 by AMG. ORO increases with AMG to similar extent as CRISPR deletion of STARD1, but in all cells. Right panels: Same cells stained for STARD1 in MITO at sites devoid of LD. AMG induces ORO-independent of STARD1 expression (Br-cAMP activation or CRISPR-deletion). Conclusion: AMG and STARD1 deletion produce similar effects by different mechanisms. Portions of C, D and E were adapted from (10).

This second role for STARD1 becomes most evident from the dramatic accumulation in lipid droplets after STARD1 deletion, mutation or functional modification (7–9) (**Figures 1A, B**). *In vivo*, deletion of any key gene underlines the role of compensatory changes during development. This is particularly evident in the adrenal gland and in the human diseases that arise from a number of loss of function mutations (11).

HDLs are spherical particles that effectively distribute CHOL between tissue locations in the blood and interstitial fluid. HDL are variable in size (diameter 7-13 nm) and composition, comprising a core of CHOL fatty acid esters (CHOL-E) surrounded by CHOL (12, 13). HDL is the prime CHOL donor for steroidogenesis, but also delivers protection from atherosclerosis through a reverse transfer (RCT) process that removes CHOL from vascular cells for subsequent liver clearance as bile acids (14).

**Figure 2:**
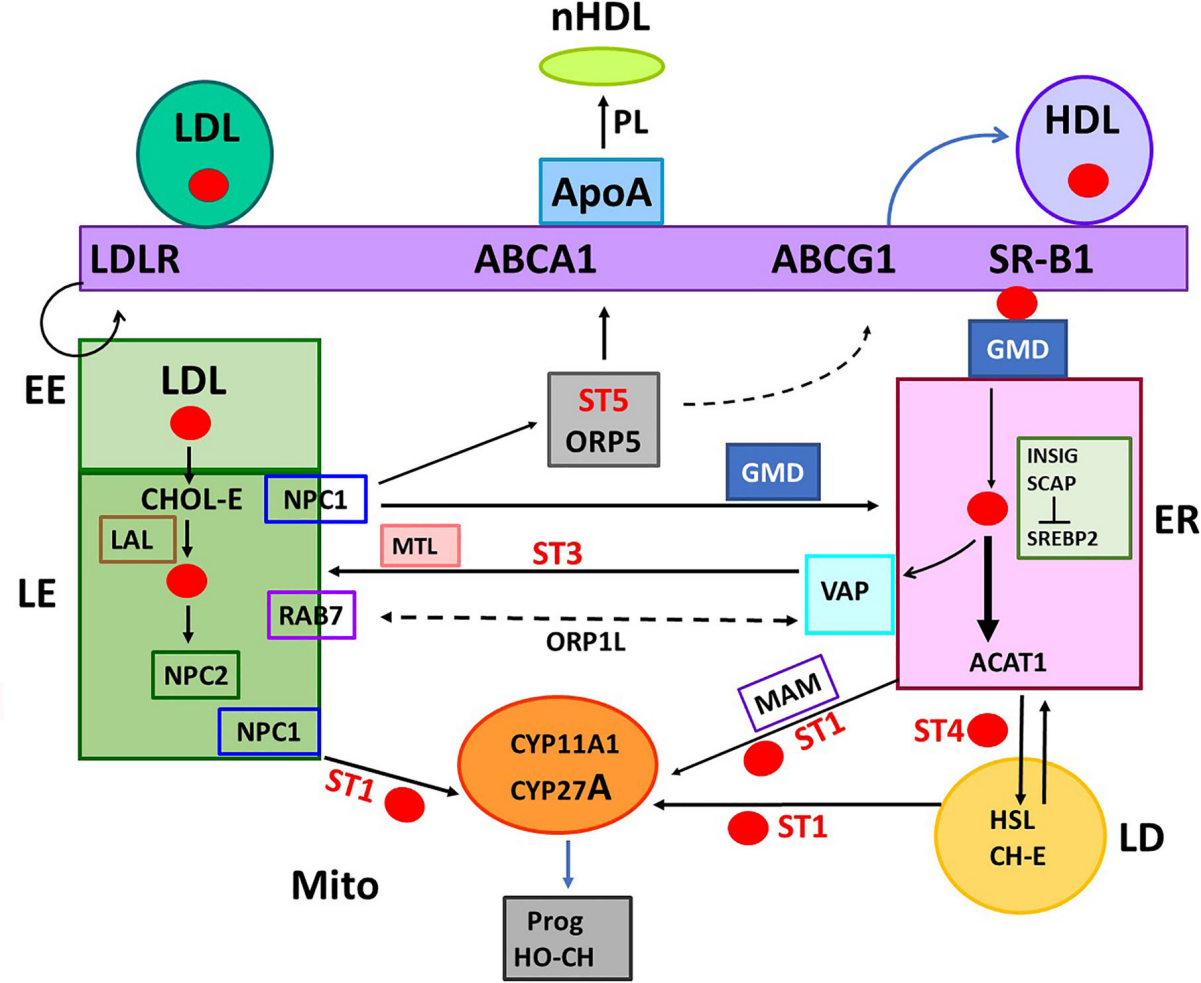
Five START proteins function in cholesterol trafficking between cell compartments and metabolism. START domain proteins function selectively in the movement of CHOL at select cell locations. STARD forms 1, 3, 4, 5 (ST 1, 3, 4, 5) complete specific inter-organelle CHOL transfer, as indicated. MTL (MENTAL) is a contact forming NTD for STARD3. GMD: GramD1B with has a central START and ER-specific C-terminal transmembrane domain. STARD proteins partner with oxysterol binding proteins. ORP1L and ORP5 each binds CHOL and HO-CHOL. Each function with extra targeting partners. Rab7 and VAP mediate MCS at LE/LY and ER, respectively. COMPONENTS OF TRAFFICKING. CHOL IMPORT: HDL, SR-B1, and GMD into ER. ACAT1 (Acyl-CoA Acyl transferase) converts CHOL to CHOL-Esters (CH-E) and mediates transfer to Lipid droplets (LD). HSL (hormone sensitive lipase) convert CH-E to CHOL for partnership with STARD1 (ST1). LDL receptors (LDLR) introduce ApoE/LDL into endosome network. LAL (lysosomal acidic lipase) releases CHOL. CHOL EXPORT: NPC1/NPC2 export CHOL from LE, mediated by ORP5. ABCA1 directs export to ApoA1 to form nascent HDL (nHDL). ABCG1 exports to HDL. CHOL SYNTHESIS: INSIG-SCAP-CHOL sensor suppresses until CHOL is very low. Then, SREBP, CHOL-dependent transcription factor, is cleaved in Golgi to activate 15 ER enzymes. CHOL RECYCLING through Autophagy: through LY-directed transfer to LE. CHOL STORAGE: as acyl esters in LD, via ER and ACAT1. CHOL MITO METABOLISM: limited by transfer from OMM to IMM, CYP11A1 produces pregnenolone. CYP27A1: produces HO-CHOL for LXRa pathways.

Macrophage, which are key agents in RCT, express STARD1 and key trafficking genes that function in adrenal cells, including SR-B1, ACAT1, CYP27A1, LXRα, and ABCA1, but lack CYP11A1 for steroid synthesis (15). Nevertheless, these same mechanisms overlap STARD1-mediated steroidogenesis in adrenal glands (3). These macrophages, which are enriched in the vasculature, accumulate Oxy-LDL and are associated with atherosclerosis, due, in part, to CHOL hydroperoxides (16). The LXRα regulation of STARD1, which is beneficial by exporting CHOL, has been modeled by the RAW 267 macrophage line (17). These macrophages take up CHOL from HDL, store CHOL as LD and export CHOL through ABCA1 and ApoA1. This export process is concluded by extraction of phospholipid from the plasma membrane (PM), resulting in the formation of nascent HDL (nHDL/pre β-HDL) (**Figure 2**). The nHDL is converted, in the blood, by LCAT to the mature αHDL form, which comprises 5% of the blood HDL content (18). It persists much more in interstitial lymphatic fluid. This cycle is optimized in KLF4-promoted M2 macrophage (19). Deletion of STARD1 results in a remarkable enrichment of the genes involved in CHOL trafficking in the adrenal and ovary (20).

Mitochondrial STARD1/CYP27A1 generates 27-Hydroxy-CHOL that activates LXRα, suppresses SREBP-mediated signaling and activates oxy-sterol binding proteins (ORPs). However, mitochondria and STARD1 are bypassed by the ER-mediated, di-iron CHOL 25 hydroxylase (CH25H). Di-hydroxy CHOL derivatives (7(α), 27- and 7(α), 25-) participate in immunity regulation (21). Locally formed bile acids (7α, 24-oic acids) are potent FXR ligands (22). CH25H is also rapidly induced by INFγ from M1-macrophage (23), thereby inhibiting CHOL synthesis. The requirement of all viruses for host CHOL leads to potent anti-viral activity through this pathway (23). 25- and 27-Hydroxy-CHOL are excellent substrates for CYP11A1, thus raising the question of whether CH25H or CYP27A1 contribute to pregnenolone formation *in vivo*. The contributions from CH25H in local macrophage are substantial in the testis after birth (24). CYP11A1 that is expressed in T lymphocytes plays a critical role in allergy responses that are blocked by AMG (25). The role of CH25HC versus STARD1/CYP27A1 needs further evaluation.

Selective lowering of SR-B1 activity in liver and adrenal cells shows that each contributes to multiple HDL effects (12). In the testis, the SR-B1/ABCA1/HDL process plays several roles (26). Steroidogenesis functions in interstitial Leydig cells, much as in the adrenal. HDL and SR-B1 function in association with STARD1 and LXRα to produce testosterone, while also recycling CHOL to nHDL. Leydig cells also synthesize bile acids, which repress steroid synthesis through activation of FXR and SHP (27). The adjacent Sertoli cells use SR-B1 to recycle CHOL through ABCA1 for delivery to germ cells. Here, nHDL may be important in local intercellular CHOL trafficking from both Leydig and Sertoli cells. In granulosa cells, specific inhibition of CYP27A1, respectively, CYP11A1 or CYP27A1 redirects STARD1 activity toward the opposite pathway. A similar reciprocity is seen in selective endocrine changes (28).

The distinctive function of STARD1 is to direct CHOL from the outer mitochondrial membrane (OMM) to the inner mitochondrial membrane (IMM), a process that is normally prevented by structural features that depend on maintenance of membrane integrity and ion gradients (29). STARD1 overcomes this barrier but is structurally flexible. This flexibility may aid the integration of CHOL transfer into rapid mitochondrial dynamics and pulsatile hormonal stimuli (30). Thus, STARD1 is controlled in a much more complex manner than other START domain proteins. STARD1 can move CHOL into mitochondria, even after removal of the N-terminal domain (NTD), which effects entry of this protein. STARD1 is active as a 1.6-kb mRNA without the additional 1.9 kb of 3’UTR in the major 3.5-kb mRNA. The dual functions of STARD1 and integration into CHOL trafficking may require these features. We address how STARD1 may partner with other START proteins and with mTORC1, the central metabolic regulator, which is activated by CHOL. We show how single molecule imaging can resolve the timing and location of the 3.5-kb mRNA delivery to mitochondria and how this process is facilitated by SIK/CRTC/CREB activity, particularly in short pulses of PKA activation (30).

An enduring question for STARD1 is whether there are additional functions for p30 STARD1 in the mitochondrial matrix. STARD1 has a key role in protecting cardiac cells from ischemia (31). CHOL enhances respiratory oxidative stress and CYP11A1 also generates oxidative stress, suggesting that such a protective role may extend to steroidogenic cells. However, this protection in cardiac cells does not require CHOL binding to the START domain. For imported mitochondrial proteins, refolding of the denatured imported protein is normally required and is mediated by matrix chaperones, notably HSP70. This has not been established for STARD1. Another family member, STARD7 (32), which transports phosphatidyl choline, refolds after import to become functional in the inter membrane space. The additional functions of STARD1 in the LXR pathway or, indeed, mitochondria in special circumstances may have different roles for the NTD and the imported START. This question will be considered further in this review.

## The Unique Role of STARD1 in Cholesterol Trafficking

### The Diverse Roles of STARD Family Members in Cholesterol Trafficking

The highest proportion of CHOL is found in the PM followed by the endosome and Golgi networks. Much lower levels are present in the endoplasmic reticulum (ER) and mitochondria. HDL and SR-B1 deliver CHOL to the PM and subsequently to the ER. The early endosomes (EE) mediate the delivery of CHOL from the cycling of LDL and LDLR to late endosomes (LE). The LE membranes fuse with lysosomes (LY) that deliver and recycle cargo provided from autophagy, including CHOL (LE/LY). The LE/LY release CHOL from CHOL-E with lysosomal acidic lipase, which functions at the low pH delivered by LY membranes (**Figure 2**). Mitochondria show proximity with ER, LE and lipid droplets (LD), but CHOL concentrates in the OMM, except when transfer to the IMM is facilitated by STARD1.

STARD3 and STARD1 have very similar structures for the C-terminal CHOL-binding domains, but are separately targeted to LE/LY and mitochondria by their distinct N-terminal domains (NTD) (33). The CHOL-binding structure, called the START domain, comprises about 200 amino acids. This structure is found in 15 genes of the STARD family. Only five of these proteins selectively bind CHOL (forms 1, 3, 4, 5, and 6) (34). The other genes have modified CHOL binding domains that target phospholipids and other long-chain fatty acid derivatives. The interaction of STARD5 with CHOL is complicated by the finding that the purified protein interacts with bile acids but not CHOL (35). However, *in vivo*, deletion demonstrates functionality as a CHOL transporter (36). Additional partners are possibly involved in the CHOL binding to the START domain.

STARD 2, 7, 10, and 11 function as phospholipid exchange proteins, with different selectivity; phosphatidylcholine (forms 2, 7, 10) and sphingomyelin/ceramide (STARD 11) (34, 37). The remaining forms use START domains in focal adhesion sites targeted by RhoGAP (STARD 8, 12 and 13) or in fatty acyl-CoA recognition sites in two hydrolases (STARD14/Acot 11; STARD15/Acot12).

Recently, the GRAMD1B gene family has been identified as a widely expressed family of sterol-binding proteins that are evolutionarily conserved in yeast (38–41). Several yeast forms play important roles in mitochondrial dynamics, supporting a role for STARD1 in this process (42).

In mouse liver, expression of STARD1 is 20-40 times lower than STARD4 and STARD5. STARD 4, 5 and 6 lack an NTD and differ primarily in their expression selectivity. STARD4 (43–46) and STARD5 (36) function in hepatocytes (36). CHOL is transferred to mitochondria for initiation of bile acid synthesis by CYP27A1 (2) and to the ER to generate CHOL-E *via* ACAT1. Deletion of STARD4 from cells results in an equivalent increase in STARD5, which otherwise shows selectivity for CHOL transfer from LE/LY to microdomains of the PM that are enriched in the CHOL export pumps, ABCA1 and ABCG1. STARD4, but not STARD5, is controlled by SREBP2, which regulates genes involved in CHOL synthesis. STARD5 is stimulated in liver by oxidative stress (36). STARD4 deletion increases CHOL-E in LD.

### Role of NTD in Determining START Functions

In the COS1 re-constitution of STARD1 activity, deletion of 62 amino acids from the NTD retains the transfer of CHOL to the recipient CYP11A1. This deletion then leaves STARD1 with only the START domain. This segment binds a single molecule of CHOL. However, this COS1 model delivers pregnenolone at rates that are far below those in steroidogenic cells, including Y-1 and MA10 cells (33, 47–50).

This Δ−62 STARD1 also enhances CHOL transfer from isolated OMM to CYP11A1, for metabolism in IMM of mitoplasts (49). This experiment employs disrupted mitochondrial membranes that provide direct access of STARD1 to the IMM and, thereby, measures CHOL transfer activity. The membrane structure of the intact mitochondria prevents such direct IMM access by OMM STARD1. The cell activity of STARD1 requires a more extensive task in inter-membrane CHOL transfer that requires mitochondrial integrity. Even mild effects of Ca^2+^ or increases in membrane fluidity enhance movement of OMM CHOL to CYP11A1 in the IMM in absence of STARD1 (51). Mitochondrial integrity is tested by support from succinate or low concentrations of isocitrate. This matrix generation of NADPH requires an intact IMM and a membrane potential that delivers ATP (51). Removal of adrenal STARD1 with a brief CHX treatment restricts ACTH stimulation of CHOL to the OMM. This CHOL is no longer accessible to CYP11A1 metabolism supported by succinate. Equivalent inhibition of CYP11A1 by AMG causes an IMM accumulation of CHOL that equilibrates with CYP11A1 and is metabolized with succinate support.

In COS1 cells, fusion of the START domain to the OMM import channel component, TOM20, retain CHOL transfer activity that is not seen for the IMM equivalent fusion protein. Comparison to CHOL trafficking experiments in other cell membranes, that we will describe, provides helpful insight. Mobilization of CHOL from the outer leaflet of the OMM to enter a region of OMM/IMM contact is necessary to reach IMM CYP11A1. This COS1 model provides important insights into what STARD1 can do but may not effectively model the higher activities of steroidogenic cells. While STARD1 reproduces S195 phosphorylation, the activation is only two-fold compared to over ten-fold in MA10 or Y-1 cells (52, 53). The 50-60 amino acid NTD of STARD1 is highly conserved and, therefore, fulfills specific functions that remain poorly understood, but will be examined below.

The NTD of STARD3 provides a very different targeting (54). The N-terminal MENTAL domain seeks out CHOL-binding regions in the LE, thus providing membrane contact sites (MCS) that can also facilitate CHOL transfer. This transfer appears to favor ER to LE. The C-terminal START domain and central FFAT sequences tether the ER through VAP proteins that seek out CHOL-rich microdomains.

GRAMD1B proteins have an NTD, which partners with the START domain to seek CHOL-enriched regions of the PM. A C-terminal trans-membrane domain anchors the protein to the ER (38, 39, 55). These START proteins that are functionally targeted contrast with forms 4, 5, and 6, which lack the NTD, but have selective activity based on surface features of the distinct START amino acids.

### Anomalous Roles for the STARD1 NTD

CHOL can be effectively transferred to the IMM without the NTD. Nevertheless, retention of the NTD suggests that an additional function is likely. STARD1 exhibits multistep intramitochondrial processing that is associated with conserved NTD cleavage sites (48). A mitochondrial membrane potential is necessary for import of both STARD1 and CHOL (29, 56). The matrix proteolysis of the imported START domain is typically slow compared to the import, but can be activated by oxidative stress (57). Mitochondria CHOL content is normally low and elevation typically lowers ATP generation (58, 59). Import of STARD1 protects against mitochondrial-mediated apoptosis, including stress responses to CHOL. Interestingly, CHOL binding to a functional START domain is not required for this protective matrix activity (31).

Deletion of STARD1 in mice results in the complete loss of steroid synthesis by the specialized steroid producing cells of the adrenal, testis and ovary (7, 8) (**Figures 1A, B**). When an NTD-deficient STARD1 transgene is restored into STARD1-KO mice (Δ−47 STARD1 mice), adrenal cells recover 40% of the corticosterone synthesis, but much less is recovered in the testis or ovary. In each tissue, the deletion of the NTD produces very high CHOL increases in LD, comparable to increases in trafficking are substantial and tissue selective. The NTD effect becomes selectively enhanced for steroid synthesis in the testis.

The accumulation of CHOL-E in Δ−47 STARD1 adrenal tissue is remarkable in view of the modest changes in glucocorticoids and ACTH. This larger effect of the NTD deletion on LD enhancement than on glucocorticoid synthesis suggests a separate role of this sequence in CHOL transfer from the LD to STARD1. A similar LD accumulation is seen in cultured cells. We used an *in situ* dual CRISPR deletion of 1.8 kb from the STARD1 gene for both adrenal Y-1 and MA10 testis cells. Thirty percent of the cells showed the excision over 12 h, which was marked by dual GFP and RFP expression (10). This deletion produces a large decrease in PKA-induced STARD1 mRNA in the marked, transfected cells that includes the time needed for degradation (**Figure 1C**). The marked cells show an increase in lipid (Oil Red O staining) within 24 h. The CHOL-E accumulations occur in LD and on the inside of LE/LY (**Figure 1D**). The CYP11A1 inhibitor, aminoglutethimide (AMG) blocks CHOL metabolism in all cells (60). AMG produced similar lipid accumulations within an hour in normal cells that are adjacent to the GFP-marked deleted cells (**Figure 1E**).

### Cycloheximide Sensitivity of Translation Coupled STARD1 Activity

**Figure 3:**
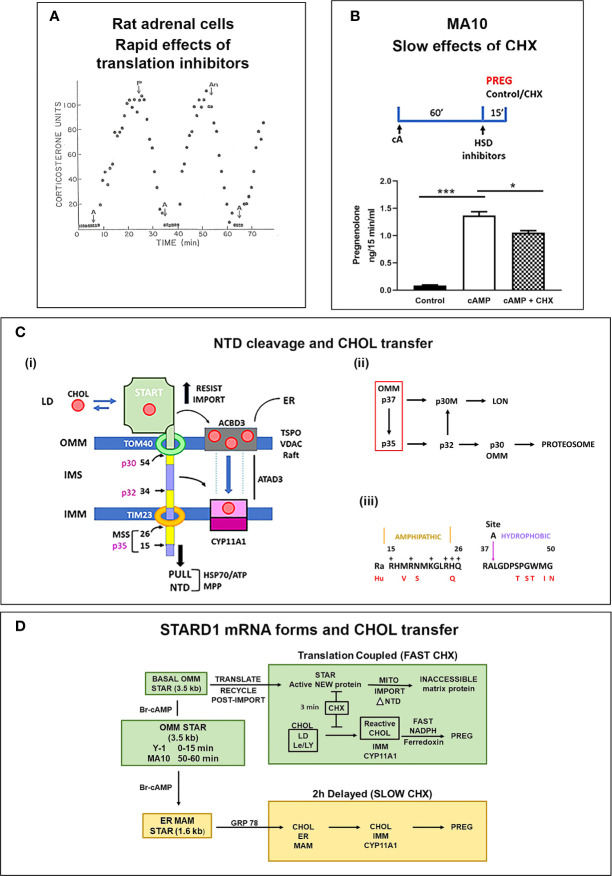
STARD1 uses two Cholesterol Transfer Mechanisms: Roles of translation and NTD. **(A)** In a fluidic model of rat adrenal cell stimulation, corticosterone secretion is increased to a peak level by ACTH **(A)** in 10 min. This secretion is reversed by translation inhibitors puromycin (P) and anisomycin (An) within 10 min. Adapted from (61). **(B)** Measurement of pregnenolone (Preg) synthesis (ng/15 min/ml), in a 15-min window, following a 60-min Br-cAMP (cA)-stimulation of MA10 cells. HSD and CYP17 inhibitors, trilostane (5 uM) and abiraterone (10 uM) were added at the beginning of the 15-min synthesis window. This activation is only modestly inhibited by the addition of cycloheximide (CHX) in this 15 min window. *p<0.05; ***p<0.001. **(C)** (i). The NTD is comprised of alternate positively charged (yellow) and hydrophobic (purple) sequences cleaved to at conserved sites by mitochondrial metalloprotease, MPP, sequentially forming p35, p32 and p30 forms or directly cleave at site 54 to release p30 to the OMM. Denaturation of the START domain pushes against protein import, the matrix HSP70 and ATP/H+ pull the NTD into the matrix. p30 at the OMM and p32 with a hydrophobic NTD in the IMS can each move CHOL into the IMM, including from TSPO rafts. (ii). The inter-conversion of cleaved forms is shown. (iii). Conserved amphoteric mitochondrial recognition and hydrophobic p32 terminal sequences. **(D)** Two distinct mechanisms of CHOL transfer (green and brown) involving fast and slow translation/import steps. The fast process involves uncoupled transcription of 3.5-kb mRNA and a translation dependent CHOL transfer. The slow mechanism involves MAM ER mitochondria contacts, translation of 1.6-kb mRNA and association with HSP78.

In a perfused rat adrenal cell model, ACTH (A) stimulates a peak of corticosterone synthesis within 15 min. Addition of protein translation inhibitors (Puromycin, P; Anisomycin, An) completely prevented this stimulation (**Figure 3A**). CHX was similarly effective in this model (61). This CHX sensitivity is also rapid in rat adrenals *in vivo* (62). This inhibition prevents the accumulation of an IMM pool of reactive CHOL and proportionate increases in CYP11A1-CHOL complexes. Mitochondrial total CHOL is increased by CHX, almost to levels produced by CYP11A1 inhibition. However, this CHOL is in the OMM and unreactive when the mitochondria are challenged with a Krebs Cycle reductant, such as succinate. This response was subsequently shown to derive from a loss of STARD1 activity (63). Mild mitochondrial disruption allows this OMM CHOL to by-pass CHX-induced STARD1 deficiency to enter the reactive pool. HO-CHOL achieves the same by-pass of the STARD1 intervention.

Y-1 adrenal cells show a maximum stimulation of CHOL metabolism by Br-cAMP within 15 min, prior to significant changes in the basic expression of STARD1 mRNA and protein. Nevertheless, CHX completely inhibited the stimulation of CHOL conversion to pregnenolone within 15 min. MA10 cells respond much more slowly. After a 60-min stimulation by Br-cAMP, transcription of STARD1 produces mRNA comparable to basal Y-1 levels. CHX treatment (15 min) immediately following stimulation decreased the rate of pregnenolone formation by only 25% (**Figure 3B**). This matches an earlier report of a 50% decrease in progesterone synthesis after 30 min (64). This slower response to CHX may reflect a slower removal of the OMM associated p37 by import.

The role of the NTD in STARD1 activity is central to the acute sensitivity of the process to inhibition of translation in Y-1 cells. In adrenal cells, *in vivo*, and in cultured Y-1 cells, STARD1 activity and steroid synthesis peak within 15 min of PKA stimulation, but are then completely inhibited by CHX within 5 min (56, 60, 62). The 37-kD pro-STARD1 also undergoes essential PKA phosphorylation at S-195 during the coupled translation-import process (47). This slow import and NTD cleavage results in a 30-kD protein in the mitochondrial matrix that no longer participates in import (48, 49). STARD1 was first identified by a coordinated ACTH or Br-cAMP stimulation of adrenal cells and rapid generation of phosphorylated p30 proteins that disappeared within minutes of CHX treatment (65, 66). This STARD1 process, from translation to matrix inactivation, is complete in about 5 min in adrenal cells, thus ensuring that a cycle of renewed translation is needed to sustain activity (56). This sequence transfers approximately 400 CHOL molecules for each cycle. The import of STARD1 is the slow step in this cycle.

### Role of the NTD in STARD1 Activity. CHOL Import Versus NTD Cleavage

During mitochondrial CHOL metabolism, p37 STARD1 NTD docks with the OMM import channel, TOM40. Pulse labeling in Y-1 cells shows that STARD1 is imported into the mitochondrial matrix, with a half-time of about 3 min, with cleavage of the NTD to a p30 form (56). This imported p30 is slowly removed by matrix proteases, with a halftime of about 2 h (52, 57). This removal is mediated by specialized matrix proteases, such as Lon (67). Interestingly, the turnover is biphasic, suggesting additional matrix effects on p30. Although forms that lack the NTD (Δ62-STARD1) function in CHOL transfer from outside the OMM, the conserved NTD still influences activity. The first function is to direct STARD1 to the mitochondrion. However, this function is repeated by a short NTD from CYP11A1, which delivers protein import in COS1 cells, but without CHOL transfer (49). However, this speed of STARD1 transfer may not be the whole story for the NTD. The STARD1 import rate in Y-1 cells appears comparable to that of this CYP11A1-STARD1 fusion protein in COS1 cells.

The STARD1 import rate and NTD size lies within the normal range for the several hundred nuclear encoded mitochondrial proteins (8-70 amino acids) (68). These proteins have N-terminal mitochondrial pre-sequences (MPS), characterized by amphipathic helices, a feature shared with STARD1 **(****Figure 3C**). The N-terminal sequence 1-14 is hydrophobic, while the subsequent 15-26 sequence is amphipathic with hydrophobic and positive sides (**Figure 3Ciii**). Cleavage close to this positive cluster corresponds to the p35 form of STARD1. The positively charged MPS passes through the IMM, *via* the TIM23 channel, empowered by an ATP-driven motor centered on matrix Hsp70. For typical proteins, this MPS is removed through cleavage by the mitochondrial processing protease (MPP). This metalloprotease accounts for the ortho-phenanthroline sensitivity of STARD1 activity (56). However, STARD1 has two further highly conserved MPS-like sequences and cleavage sites at positions 34/35 (Site A) and 54/55 (Site B). Three cleavage STARD1 forms are repeated in adrenal, testis and non-steroidogenic, COS1 cells, as well as in mice, rats, cattle and humans (**Figure 3C**) (48). In mice, the A and B sites correspond to, respectively, p32 and p30 STARD1. The A and B positive clusters match sequences that can enhance passage through the TOM40 and TIM23 channels (68).

The STARD1 import is potently inhibited by CCCP, which suppresses membrane potential *via* an ATP-dependent proton pump (29, 56). Nigericin, which targets this pump with less effect on membrane potential, also lowers both protein import and CHOL transport. This selectivity suggests that CHOL and protein transport depend on ATP-dependent proton export to balance the positive charges in the imported NTD.

The pull is provided by the ATP-dependent motor of the HSP70 complex, which sequesters the MPS of the NTD. This pull is opposed by the necessity to unwind the START domain. The START domain also interacts with CHOL from LD or LE/LY through a 1:1 complex prior to NTD import (69). During steroidogenesis in adrenal cells, this complex is transient, since each STARD1 enters the mitochondrion within a few minutes in a denatured, inactive form. Nevertheless, several hundred CHOL molecules transfer in and out of the START domain in the 3 min between formation and unwinding (56). CHOL may move from the LD or LE/LY to a CHOL-receptive raft in the OMM that is made accessible through contacts with ER and IMM (70, 71).

Typically, there is a correlation between weak binding to the START domain and low CHOL metabolism (69). Several of these mutations are found close to the PKA phosphorylation site, S195. Mutations at S195 and phosphorylation also affect CHOL binding. PKA activation has little effect of the pattern of STARD1 import (52, 53). Reversible START folding is aided by the flexibility of this molten-globule structure (72). A transient START conformation in the rapid import of Y-1 cells may be appreciably different from a stable p37 structure. Weaker CHOL binding is compatible with the fast association and dissociation of an effective exchange mechanism.

There appears to be alternative ordering of MPP proteolysis of the NTD upon entry into the matrix. Mutations at the A and B cleavage sites, which are confirmed with peptide mass spectrometry, produce very different changes in the intermediate STARD1 forms (48). Mutation at site A strikingly removes p32, attenuates p30 and increases both p37 and p35. This suggests that site A cleavage rapidly removes p35 to form p32, prior to a slower release of matrix p30 (**Figure 3Cii**). Mutation at site B attenuates p30 and increases p37 but has little impact on p32. A complete pull by the HSP70 motor to site B, while retaining a folded START, generates a p30 that remains outside the OMM and is turned over by the cytoplasmic proteosome. The same HSP70 activity on an unfolded START favors import into the matrix (57). CHOL availability for START complex formation and S195 phosphorylation should affect this balance.

These considerations suggest that p37 and two intermediates, p30(OMM) and p32, may each function in steroidogenic mitochondria. A fourth contributor, matrix p30, may contribute to some kind of STARD1 activity, if there is re-folding of the START domain, a process that is initiated by HSP70 for essentially all normal imported mitochondrial proteins. Their contributions may depend on cell type, mitochondrial location and timing (70). The p32 form (Δ-39 STARD1) is of particular interest. The N-terminal sequence consists of uncharged side chains, of which four are fully conserved (LGxxxPxxWxx), adjacent to the most positively charged cluster in the NTD (QxxRRR) (73) (**Figure 3Ciii**). The signaling capacity of such lipophilic transmembrane peptides has recently been established (74).

The NTD processing in STARD1 is similar in the PC transfer protein, STARD7 (32). This form has a START domain that is modified, like STARD2, to accommodate PC, but with a long NTD that is targeted for mitochondrial import. STARD7 shows an N-terminal MPS cleavage site, like STARD1, with a second specialized protease site in the center of the NTD that is equivalent to site A in STARD1. This NTD fully imports STARD7 into the inter-membrane space (IMS) (32), where the START domain mediates OMM to IMM PC exchange. Cleavage of STARD7, exclusively at the central site, returns the shortened STARD7 to function outside the OMM. STARD1 is also appreciably detected by electron microscopy in the IMS (57).

### Cholesterol Transfer to STARD1 and Mitochondria From ER Through MAM Sites

In MA10 cells, STARD1 participates in a second CHOL transfer process that is associated with a complex that forms in ER-OMM contact sites (MAM) after about 2 h (**Figure 3D**). The ER strongly influences mitochondria through these MAM sites, which form up to 5% of the mitochondrial surface (75). Mitochondria are in a constant state of fusion and fission that is dependent on the ATP energy state (76), which is integrated with these MAM contacts (77). This process substantially impacts STARD1 activity (78).

STARD1-mediated CHOL transfer in MA10 cells has been attributed to large complexes that form slowly at these MAM sites after stimulation by PKA (70, 79). A 450-kD multi-protein complex, marked by STARD1, has been extracted from MAM sites with digitonin. This complex includes the sigma-factor from CHOL-enriched ER caveolin rafts, the ER stress protein, GRP78 and OMM VDAC2. GRP78 presence appears to mobilize STARD1 into this complex (80). This complex becomes apparent only after about 2 h and probably does not contribute to the appreciable activity that is seen within 30–60 min of stimulation **(****Figure 3D****)**.

These MAM sites are important in the fusion-fission cycle of mitochondria (76). The relationship of these fast and slow transfer processes to mitochondrial cycling remains to be resolved. The giant mitochondria formed in this cycle are a feature of adrenal cortex cells. Mitofusin2, a GTPase, selectively enhances MAM activity and STARD1 participation in CHOL metabolism (37, 78). Another protein, FATE1, attenuates MAM contacts and restricts both Ca^2+^ transfer and steroid synthesis (81). STARD1 activity in MAM sites also depends on specific S232 phosphorylation by OMM-associated, Erk kinase (37, 78, 82), and partnership with TSPO (71).

### Partnership of STARD1 With TSPO

TSPO is a widely expressed 18-kD OMM protein that comprises 5 α-helices, of which the C-terminal segment contains a CHOL recognition amino acid consensus (CRAC) (83). TSPO activates the OMM voltage dependent anion channel (VDAC) that is stimulated (benzodiazepines, isoquinolines) or inhibited (flunitazepam) by drugs that have equivalent effects on CHOL metabolism. Photo-crosslinking of MA10 mitochondria with an azido-CHOL derivative generated an 800-kD multi-protein complex (84, 85). This association has little overlap with the 450-kD MAM cluster and, notably, does not contain STARD1 (70). Digitonin extraction and this photo-crosslinking should show selectivity for CHOL-rich membrane segments. Both clusters include MAM markers but have different components. The 800kD cluster contains OMM and IMM proteins, including TSPO, VDAC1, the PKA docking protein, ACBD3, and two 14-3-3 phospho-protein docking proteins, the ATPase, ATAD3, and CYP11A1. The variant ATAD3 captured here has an extended NTD that inserts in the ER. The remainder of the protein bridges the OMM and IMM. Mutation of the CRAC sequence greatly decreases CHOL binding to TSPO. hCG increases the 800-kD complex in parallel with CHOL metabolism over the initial 2 h (71).

There has much controversy over the role of TSPO in mitochondrial CHOL transfer (86). SF1-Cre conditional deletion of TSPO resulted in offspring with TSPO deletion from testis and adrenals. Significantly, testosterone production in the testis was not affected. Basal adrenal corticosterone production was extensively suppressed (87). This resistance confirms a previous finding that testis can produce testosterone without TSPO (88). In fact, the prime difference occurs for the extra steroidogenesis provided by ACTH stimulation. In rats, TSPO has been fully suppressed with a Zn finger nuclease deletion of TSPO from fertilized oocytes (89). This resulted in a decrease in ACTH-induced corticosterone in half the rats and a more complete suppression of basal testosterone. Lipid droplets increased in the testis and adrenal, much as with STARD1 deficiency. However, MA10 cells deficient in TSPO show increased fatty acid oxidation and ROS production, in parallel with increases in the PM fatty acid transporter, CD36, several genes that promote fatty acid oxidation, and UCP2 (90).

Investigations directed to mitochondrial function rather than steroidogenesis emphasize the key interaction with VDAC1, which mediates ATP transfer across the OMM. TSPO promotes oxidative stress by suppressing ATP (91). The mitochondrial functions of TSPO, VDAC1, are CHOL dependent and occur in non-steroidogenic cells.

The data for TSPO disruption in mice and rats points to a context-dependent contribution to steroidogenesis that is more frequently found in the highest activities seen with ACTH-induced adrenal compared to the basal metabolism or with the lower rates in the testis. STARD1 activation of CHOL metabolism can clearly function without TSPO. Even modest effects on mitochondria enhance OMM-IMM contacts that probably underpin mitochondrial CHOL transfer. The regulation of local ATP movement through VDAC1 and effects on metabolism and ROS can potentially contribute to STARD1 activity and cannot be overlooked. Contributions from the NTD and different forms of STARD1 should be viewed in the same light.

### STARD1, CYP11A1, and Reactive Mitochondrial Cholesterol Pools

CHOL metabolism by CYP11A1 is supported by NADPH that is delivered by an iron-sulfur protein, ferredoxin (adrenodoxin), and a flavoprotein reductase (1). CYP11A1 has a very high affinity for CHOL and fast turnover for the three concerted oxygenase cycles that generate pregnenolone. CYP11A1 is located on the inner face of the IMM, where it surprisingly impacts mitochondrial morphology (92). Much less is known about CYP27A1, including the relative levels, location and activity (93). These two pathways can function competitively (28). Side chain HO-CHOL derivatives by-pass the constraints on CHOL transfer that are removed by STARD1 (56). The CHOL affinity and activity at CYP27A1 are each modest compared to CYP11A1 (1, 93). Therefore, more IMM CHOL and STARD1 activity may be needed to maintain CYP27A1 metabolism and induction by LXRα. This process is also effectively coupled to hormone-sensitive lipase (HSL) activity in LD (2, 94). Little is known about whether an active START domain from p30 contributes to CYP27A1 activity. We have noted the difficulties in defining the roles of forms 3, 4 and 5 in CHOL trafficking or STARD7 in mitochondrial PC transfer.

Adrenal mitochondria have a specialized zone of mobile CHOL in the IMM that equilibrates with CYP11A1. This CHOL pool is maximized *in vivo* and in cultured adrenal cells when CYP11A1 is inhibited by AMG (60, 62). This reactive IMM CHOL pool is enhanced by ACTH, up to about 5–10 molecules for each CYP11A1, and is metabolized *in vivo* and in cultured cells or isolated mitochondria within 5 min. Rapid isolation of mitochondria from live rats reveals that this pool of reactive CHOL is then very small but is enhanced by the rapid depletion of oxygen after adrenal excision (95). A second pool of mitochondrial CHOL, which we previously attributed to transfer from OMM CHOL, is identified by a slow transfer and metabolism by IMM CYP11A1 that is linear for 30 min (96). This transfer can now be attributed to STARD1. Re-examination of this slow CHOL transfer shows differential stimulations by low levels of buffered Ca, addition of GTP and removal by in vivo CHX treatment (STARD1).

In cultured bovine adrenal cells, the AMG-induced pool of reactive CHOL that is rapidly converted to pregnenolone doubles in 2 h, in parallel with an increase in mitochondrial CHOL and CYP11A1 complex formation (60). Each declines extensively over the next 12 h suggesting a protective IMM adaptation to the toxicity of accumulated CHOL. Such changes warrant further examination in the light of our expanded knowledge of different forms of STARD1 and of partnership with TSPO, MAM complexes and other newly recognized mitochondrial features.

An increasing number of cell types have been identified that express STARD1, but lack CYP11A1 (15, 17, 31). This uncoupling is seen as STARD1 entry precedes steroid production in the fetal testis (97).

## Transfer of Cholesterol to STARD1 from Multiple Trafficking Sites

### CHOL Transfer From HDL to ER

CHOL from HDL is delivered by SR-B1 to the PM and subsequently to the ER by GramD1B. When CHOL is depleted by methyl β−cyclodextrin (MCD), the GramD1B/AsterB protein is distributed as speckles throughout the ER, whereas CHOL loading redistributes GramD1B to the ER adjacent to the PM (38). A pairing of NPC1 in LE/LY with GramD1B may function similarly to transfer CHOL from endosome vesicles to ER (39).

The PM has the highest CHOL content among cell membranes. Specific bacterial proteins selectively sequester CHOL and, thereby, resolve PM content into freely available, partially available sphingomyelin—associated and unavailable (98, 99). The freely available pool is bound by a fluorescent derivative of the bacterial sterol-binding protein, anthrolysin-O-D4 (98). This reagent prevents CHOL that is transferred by GramD1B and accessed by SR-B1 from entering the ER from the PM. Anthrolysin-O-D4 sequestration of PM CHOL strongly stimulates the SCAP/SREBP homeostasis (99).

Cellular CHOL homeostasis is maintained in the ER by adjustment of de-novo CHOL synthesis. This homeostasis, nevertheless, balances import and export processes. This synthesis adjusts, through membrane contacts, to CHOL levels in the LE/LY, which accumulates CHOL from both LDL endocytosis and from the internal autophagy of cell membranes (100, 101). CHOL synthesis from acetyl-CoA occurs through a set of 15 genes controlled by SREBP2. A further 9 genes convert acetate to oleoyl-CoA. These fatty acid reacts through ACAT1 to generate CHOL-E. These fatty acid genes are regulated by SREBP-1c (102). LDLR and STARD4, but not other STARD genes, are also integrated into the SREBP network.

### Cholesterol Transfer From STARD1 and Mitochondria From Lipid Droplets

The ER is comprised of a tubular network, with distinct specialized activities in the lumen and on the cytosolic surface. The ER plays a crucial role in lipid synthesis and delivers CHOL to LD, which provides a reservoir for excess triglycerides (TG) and CHOL-E (103–105). LD bud from specialized segments of the ER as they accumulate neutral lipids between the phospholipid bilayers (105). LD maintains an outer layer of phospholipids and specialized proteins, including perilipins and SNARE proteins (106, 107). Equilibration of ER CHOL with LD CHOL is additionally determined by sterol O-acyltransferase and HSL, two enzymes that liberate CHOL from the esters (104).

The steroidogenic cells and hepatocytes have specialized needs for LD, which is reflected in the differences in the content of CHOL-E and TG (108). Perilipins deliver selectivity for forming LD with energy-storing TG and steroid precursor, CHOL-E, respectively. One SNARE protein, SNAP23, may enhance interaction of LD with mitochondria (106, 107). TG droplets enlarge under control of fat-specific protein 27, whereas the CHOL-E droplets remain small. These droplets are mobile through contact with microtubules, facilitated by SNARE proteins and dynein motors.

*In vivo* stress enhanced both the reactive IMM pool of CHOL in isolated adrenal mitochondria and the external CHOL transfer across the OMM/IMM barrier to CYP11A1. These experiments also established a role for GTPases, specifically in the external transfer as evidence by GTP activation and reversal by non-hydrolysable, GTP*γ*S (96). CHOL metabolism has now been reconstituted with recombinantΔ−62 STARD1, steroidogenic mitochondria and an external source of CHOL. The contributions from LD proteins are of major importance. This model was much improved by the addition of seven recombinant SNARE proteins and Δ−62 STARD1 (109). STARD1 and the SNARE proteins effectively increased CHOL metabolism and reactive CHOL. However, this work leaves open the question of whether the contributions of the NTD or ongoing translation of STARD1 protein can be captured in this way.

SIK1 is an intriguing regulator of SR-B1. Stimulation is effected by direct association and phosphorylation at S496 (109). This is one of several activities of SIK1 at the PM, including direct activation of Na/K ATPase and suppression of Par3, a regulator of Gap junctions (110). SIK1 is active in the nucleus as a STARD1 suppressor when unphosphorylated at S577 (111), but has an alternative activation at the PM by CaMK1. Here, coupling to Na/K ATPase elevates Ca^2+^ and SIK1 activation through phosphorylation at S322 (110, 112). CaMK1 also replaces PKA to directly activate STARD1 in the angiotensin regulation of adrenal glomerulosa cells (66). This Na/Ca activation of SIK1 is optimal at low ATP, notably during adrenal hypoxia (95).

LD provide a site to remove excess CHOL from the ER (103) by means of CHOL-activated, ACAT1, a membrane-spanning ER enzyme (106). The CHOL-E shows selectivity for long chain (C18-C22), unsaturated fatty acyl-CoA derivatives, providing an enriched source of these active fatty acids. CHOL is released from the esters in adrenals and testis by activation of HSL, *via* PKA-stimulated phosphorylation (S563, S565) (94). Inhibition of HSL by CAY10499 lowers STARD1, paralleling effects of HSL deletion (113). STARD1 with CYP27A1 activates LXRα to enhance both STARD1 and the CHOL export pathway through ABCA1.

### Cholesterol Transfer to STARD1 and Mitochondria From Late Endosomes

CHOL transfer from the LE/LY to STARD1 and mitochondria is less resolved than for LD. There is, however, increasing evidence for contacts between LE/LY and mitochondria (45). In steroidogenic cells, the synthetic NPC1 ligand, U18666A, inhibits steroid synthesis, thus implicating NPC1-mediated transfer in the STARD1 activity at the mitochondria (114). The *in situ* CRISPR removal of STARD1 not only increased LD, but also deposited lipid on the inner surfaces of large vesicles (**Figure 1D**), typical of LE/LY marked by LAMP1 (10, 115).

**Figure 4:**
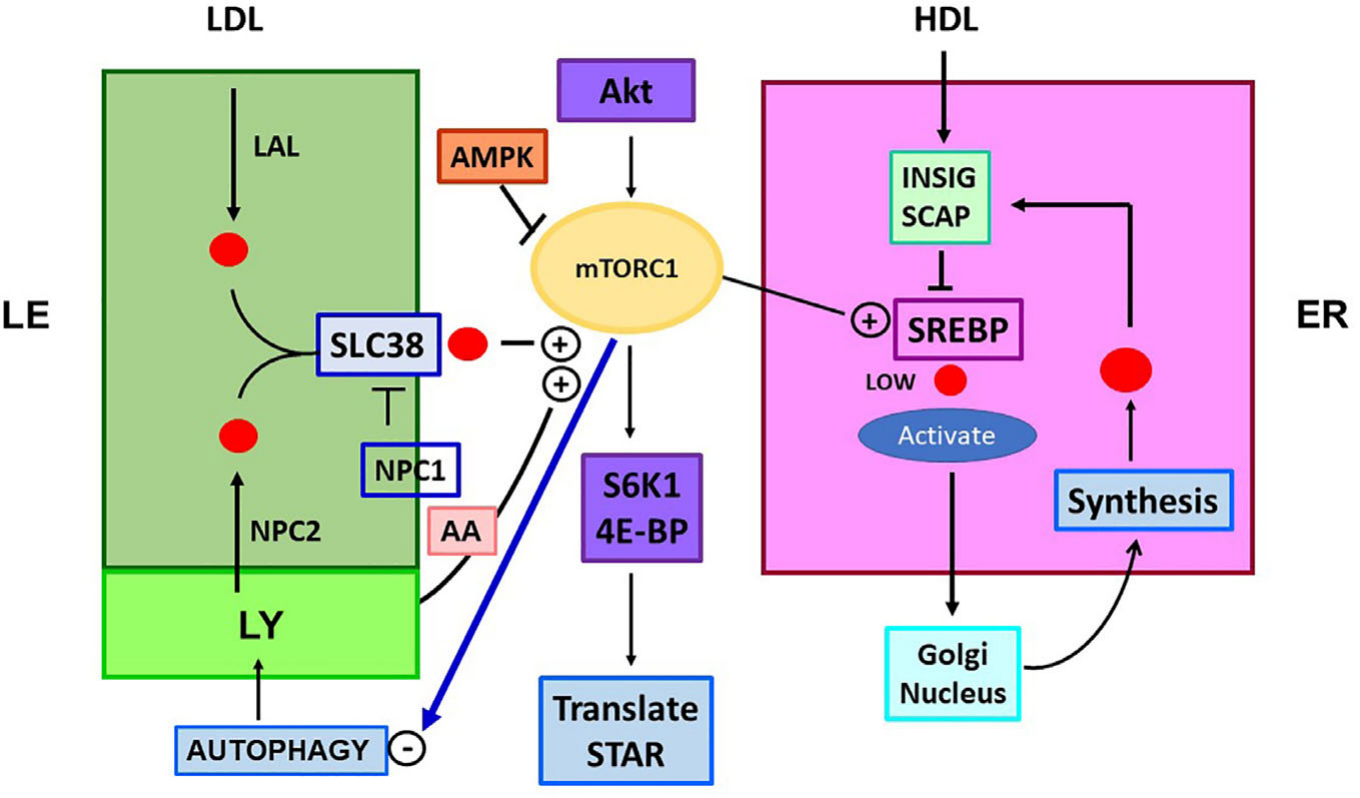
Role of mTORC1 in cholesterol trafficking and synthesis. Is there a link to STARD1 activity? The mTORC1 regulatory complex controls TOR1 kinase through multiple associated proteins. This activity is independently stimulated through LE/LY CHOL, which complexes with SLC38A9 (SLC38) and by amino acids (AA; Arg, Leu, Glu), each effecting association of mTORC1 with the lysosome surface. CHOL in LY/LE that forms the SLC38A9 complex derives from endocytosis of LDL/LDLR, delivered as CHOL-Esters. Lysosomal Acidic Lipase (LAL) releases free CHOL. CHOL is also delivered by lysosomes, including from autophagy recycling. NPC inhibits this activation directly and by CHOL depletion through export. mTORC1 is stimulated by insulin and growth factors that function through Akt and from diet via suppression by AMPK. mTORC1 is suppressed by ER stress processes (ATF6/PERK). TOR1 kinase activity increases CHOL synthesis, by lowering negative INSIG effects on SREBP2 activation by SCAP, when ER CHOL is low. TOR1 kinase suppresses autophagy and LY fusion to LE, but increases protein translation, via phosphorylation of ribosome S6K1 and initiation factor, 4E-BP. These effects on translation may extend to control of translation coupled STARD1 activity.

Fibroblasts that do not express STARD1 distribute CHOL to mitochondria from LE/LY, but only when NPC1 is deleted (116, 117). This process depends on STARD3. In cells that lack STARD1, additional removal of the START domain from STARD3 causes loss of steroid synthesis, accompanied by CHOL accumulation in LE/LY. However, mutation of STARD3 in mice that express normal STARD1 had minimal effect on CHOL metabolism or distribution (118, 119). Thus, STARD3 may route CHOL to mitochondria in absence of STARD1 but does not function significantly as a partner. Inhibition of NPC in MA10 cells may, therefore prevent direct transfer of CHOL from NPC to the START domain of STARD1, equivalent to the transfer to the START domain of GramD1B (**Figure 4**).

Evidence for contacts between LE/LY and mitochondria is emerging (115, 120). An alternative transcript of the mitochondrial fission GTPase, DRP1, marks sites of LE/LY mitochondrial contacts in neurons (115, 120). LE/LY, marked by Rab7, commonly co-localize with mitochondria, ribosomes and mRNA that LE/LY, marked by Rab7, commonly co-localize with mitochondria, ribosomes and mRNA that generate proteins. These interactions match those expected for STARD1 translation coupled CHOL import.

## Cholesterol Homeostasis and Transfer Between ER and LE/LY. Links to mTORC1 and STARD1

### Cholesterol Trafficking Between the PM and ER in Relation to Cholesterol Homeostasis

CHOL synthesis depends on free levels detected in the ER. The conversion of Acetyl-CoA to CHOL and to C-20 fatty acids is coordinated by the regulation of all CHOL genes by SREBP2 and all fatty acid genes by SREBP-1c. These regulators are transmembrane proteins with an active NTD, which is released, as a transcription factor, by cleavage in the Golgi. SREBP are prevented from activation by SCAP and INSIG, which together retain SREBP in the ER (102). At low CHOL, SCAP changes conformation and dissociates from INSIG. Homeostasis functions when CHOL is at or below 5% of total ER lipid, a level far below the 45% content of PM. In this 5% range, CHOL proportionally enhances the binding of the SCAP-INSIG-SREBP2 complex to the ER. As CHOL declines, the complex is released for activation by Golgi proteases. This SCAP-SREBP complex is transferred to Golgi as vesicles, mediated by the COPII vesicle assembly.

HO-CHOL (principally 25-and 27-) play major roles in the suppression of CHOL synthesis. In addition to their roles as activators of LXRα, they enhance this INSIG interaction, thus further suppressing synthesis. HO-CHOL also rapidly inhibits the pathway by binding to a sterol-sensing domain of HMGCoA reductase, which becomes destabilized, thereby removing mevalonate, a multi-pathway intermediate. 27HO-CHOL forms through STARD1 and CYP27A1, whereas 25HO-CHOL by-passes mitochondria, *via* the specific generation in the ER by CH25H (121).

### Cholesterol Transfer Between Late Endosomes, PM, and ER

CHOL is imported into the endosome network by LDL/LDLR endocytosis. Release of LDL delivers CHOL-E that are converted to free CHOL by lysosomal acidic lipase in the LE/LY (**Figure 2**) (40, 55, 100, 122). There is a countering export process to the PM, directed by three CHOL binding proteins: the transmembrane, NPC1; the smaller intra-organelle partner, NPC2, and the cytosolic oxysterol-binding protein, ORP5. Deletion of each protein causes CHOL accumulation in LE/LY (2, 55, 101), thus implicating these proteins in a joint export process. STARD5 participates in CHOL export to the PM (36).

A key issue is the connection of CHOL levels in the LE/LY to the homeostasis of CHOL synthesis in the ER (100). Three types of connection between these organelles have been characterized. When ER CHOL falls, transport from the LE/LY can more rapidly restore homeostasis than new synthesis. Excess CHOL transfer can be accommodated by ACAT1 and LD. NPC1 mediates this transfer by concentrating at MCS, where there is enrichment of GramD1B in the ER (55). NPC1 effects on CHOL content in LE/LY are altered by the inhibitor, U18666A, which selectively decreases these contacts with the ER. STARD3 mediates the opposite transfer by taking up CHOL from VAP proteins in the ER to sites in the LE/LY that are recognized by the NT MENTAL (MTL) domain (54). A protein related to ORP5, ORP1L (122, 123), controls the positioning of LE/LY in a way that depends on synergistic binding of CHOL and the phosphoinositide, PI(4,5)P2 (123). ORP1L has an N-terminal ankyrin repeat that connects with the GTPase, Rab7, and the microtubule network (122). When CHOL is low, the conformation changes such that ORP1L binds the ER VAPs, and enhanced ER-LE contacts, through Rab7, cause microtubule association and movement to proximity with the PM.

CHOL transfer can occur through vesicular transport processes that depend on dynamic GTPases (100, 124–126). Vesicle transfer that activates is involved in the ER-Golgi transfer of CHOL biosynthesis in the cycling of endosomes. These processes, which are directed by microtubules, depend on attached ATP-dependent dynamin complexes operating on microtubules. This transport is removed by inhibitors of ATP production and by microtubule disruptors. CHOL trafficking from ER and LE/LY to Golgi and then to PM involves microtubules, like the Rab7 movement in conjunction with ORPL1 (122). The STARD1-dependent conversion of CHOL to pregnenolone in mitochondria is extensively and rapidly inhibited by both microtubule and microfilament disruptors (61, 127).

### The Metabolic Regulator, mTORC1, Plays a Central Role in Cholesterol Regulation

The multi-protein complex, mTORC1, impacts CHOL trafficking and signaling in multiple ways. This complex mediates the inhibitory effects of Rapamycin on many cell functions. The multiple interacting factors that comprise this complex control the activity of TOR kinase (128). **Figure 4** shows activation by Akt, which mediates signaling from insulin, EGF and related growth factors that can also stimulate STARD1. Conversely, deprivation of energy, seen as a loss of ATP, elevates AMPK, which suppresses factors that control TOR kinase. mTORC1 activates SREBP2 activity through phosphorylation and suppression of the major lipid regulator, Lipin, which enhances release of the active transcription factor. SREBP1 is separately activated by S6K (129). mTORC1 also suppresses CHOL gain through the re-cycling of autophagy.

This is part of a broad stimulation of lipogenesis linked to tissue growth (130). The activation of SREBP2 and ER CHOL synthesis modulates a general lipogenic response to growth signals, which is promoted when CHOL is available in LE/LY from diet (LDL) and autophagy recycling. A second potential connection to STARD1 derives from stimulatory effects on protein translation through phosphorylation of S6K1 and 4EBP1 (122). This connection has the potential to affect translation coupled STARD1 activity in adrenal cells. In testis and adrenal, CHOL metabolism has been examined in mice that lack ATG5, a protein that mediates these autophagy fusions (131). This autophagy contribution to mitochondrial CHOL metabolism increases with age. This contribution to steroid synthesis connects to STARD1 through changes in SR-B1.

A large part of mTORC1 activation depends on physical attachment to LE/LY, which is activated in proportion to the CHOL content of the LE/LY and the release of three sensor amino acids; glutamine, arginine and leucine (132, 133). The CHOL content of the LE/LY derives from endocytosis of LDLR (134) and lysosomal delivery from autophagy (**Figure 4**). This autophagy contribution is subject to strong inhibition by mTORC1 kinase of the fusion of LY and LE (135). The activation derived from CHOL in the LE/LY is mediated by SLC38a9, which directs contacts of mTORC1 to the outer LE surface (**Figure 4**) (58). CHOL-bound SLC38a9 on the LE/LY surface binds RAG GTPase components of mTORC1, which then activate mTOR kinase (128, 133, 135). When CHOL is low in the LE/LY, SLC38a9 association with NPC1 prevents the binding of mTORC1 and the accompanying activation. Conversely, when CHOL is high in the LE/LY, the activated mTORC1 limits further CHOL delivery from autophagy.

ER stress suppresses mTORC1 through the UPR activation of PERK, a kinase that inhibits general protein translation; XBP1, which removes misfolded proteins; and ATF6, which stimulates chaperones that facilitate protein folding. One such chaperone, GRP78, directs the transfer of STARD1 into MAM structures (80) (**Figure 3D**).

## Complexity of STARD1 Transcription in Relation to Cholesterol Transfer Mechanism

### *In Vitro* Limitations, Pulsatile Activation and Single Cell Resolution

STARD1 expression and stimulation of CHOL metabolism in adrenal and testis cells have mostly been studied with constant cAMP activation of PKA. *In vivo*, this constant PKA stimulation is replaced by 15-min pulses of ACTH in the adrenal cortex or of leutinizing hormone (LH) to the testis. The adrenal pulses occur at approximately hourly intervals, dependent on the level of stress, and exhibits strong diurnal variation (136, 137). For adrenal glands, *in vivo*, single ACTH pulses generate primary STARD1 transcripts that retain introns, but little mRNA. These *in vivo* findings are replicated for STARD1 in both Y-1 adrenal and MA10 testis cells. The brevity of the adrenal pulses matches the rapid cycling of STARD1 in the translation coupled adrenal CHOL metabolism that has been studied *in vitro* by perfusing primary adrenal cells with ACTH and translation inhibitors (138) (**Figure 3A**).

### Multiple Transcription Factors in Relation to the Time of Stimulation and Activator

ACTH and LH elevate cAMP through Gα stimulation of adenylate cyclase. This activity is uniquely enhanced for the MC2R/ACTH receptor by the interacting membrane partner, MRAP (139). ACTH and LH also activate STARD1 expression through Ca^2+^ and the PKC/MAP kinase pathway, when stimulated by phorbol esters (TPA) (5). Stimulation of voltage-dependent Ca^2+^ channels, in combination with EPAC1, contributes slower changes to adrenal cells (5, 140). Phosphodiesterases (PDE4, PDE8) deliver local regulation through removal of cAMP (141, 142). The importance of PDE4 and PDE8 in the control of adrenal activity is shown by the prevalence of loss of function mutations in the adrenal hyperactivity of Cushings Disease (143). Regulatory subunits of PKA deliver spatial selectivity for the catalytic subunits (144).

### Novel Uncoupling of Elongation and Splicing During STARD1 Transcription

**Figure 5:**
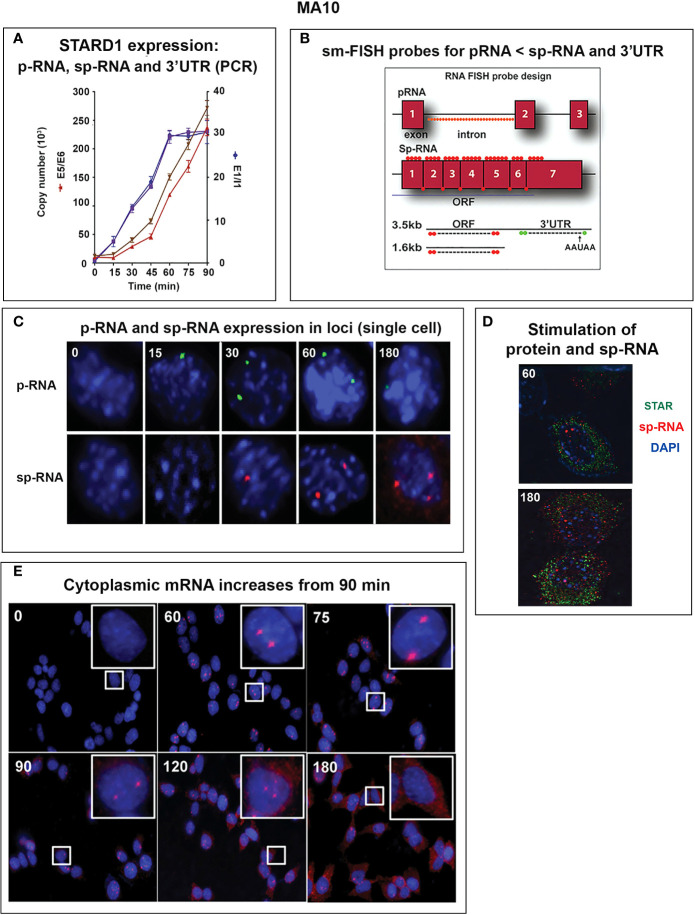
Delayed splicing and asymmetric STARD1 expression at loci in MA10 cells. **(A)** Immediate increase in copy number for p-RNA at introns 1 and 6 reaches a steady state after 60 min of 30,000 copies (blue, right scale). Spliced transcripts (ORF) show delayed increases in sp-RNA (red, left scale). Primers measuring the extended UTR (brown, left scale) also show delay. **(B)** sm-FISH 40 oligomer sets for assessment of STARD1 p-RNA locus, sp-RNA/mRNA and extended 3’-EU of the 3.5-Kb mRNA. **(C)** Time course for asymmetric appearance of p-RNA (green, upper) and slower sp-RNA (red, lower) at STARD1 loci. **(D)** Stimulation of mRNA measured by sm-FISH (red) and accumulated STARD1 protein (antibody, green) in mitochondria. **(E)** sm-FISH detection of sp-RNA at loci and cytoplasmic mRNA at 0, 60, 75, 90, 120, and 180 min. Parts of this figure were previously published (145, 146).

STARD1 has seven exons, separated by six introns. Primary RNA that retains the introns are measured by primers located at the beginning of introns 1 and 6. Spliced RNA, before and after processing to mRNA, is determined by primers that bridge adjacent exons. Copy numbers are determined, here, by specific reverse transcription and reference to corresponding DNA blocks (**Figure 5A**). The long terminal exon 7 produces alternative polyadenylation products that have been analyzed by PCR at different 3’UTR positions (**Figure 5B**).

In MA10 cells, where basal STARD1 is essentially absent, Br-cAMP initially generates primary transcripts that retain all the introns (p-RNA; E1/I1, blue line) (**Figure 5A**). The first and last introns appear together, an indication of fast, continuous elongation of the primary transcript. A steady state is reached after 60 min. Splice removal of the introns (sp-RNA; E5/E6, red line) is delayed for about 15 min and a secondary surge occurs after 45 min. The 60-min p-RNA steady state is outpaced by sp-RNA at this time by about 5-fold. This PCR analysis cannot distinguish RNA at the nuclear loci compared to cytoplasmic mRNA. There is significantly more RNA from the end of the 3’UTR consistent with contributions from both primary transcripts that are at the gene loci and mRNA.

To understand the relationship between STARD1 mRNA and CHOL metabolism, we developed the means to separately visualize individual molecules of each form in single cells (146). sm-FISH probes target 800 base sequences with 40 contiguous fluorescent 20-base oligomers (**Figure 5B**). The probe sets have sufficient affinity and combined fluorescence to detect even a single transcript of p-RNA, sp-RNA or 3’-EU at the loci. STARD1 protein is localized in mitochondria by immunohistochemistry (147, 148).

**Figure 7:**
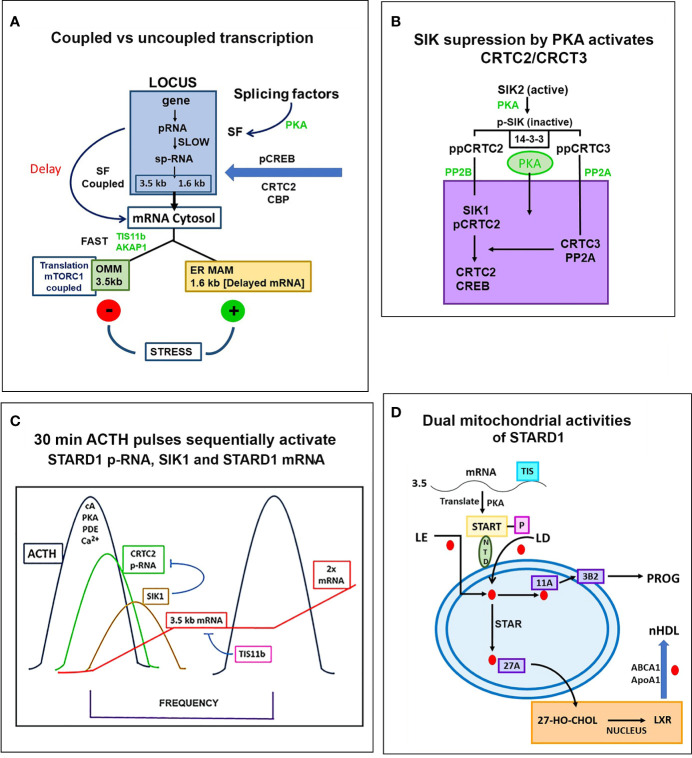
Special role of SIK and CRTC forms in the PKA stimulation of STARD1 RNA. **(A)** Slow splicing and alternative polyadenylation. Uncoupled generation of p-RNA is indicative of the delayed availability of splicing factors. Selective roles of TIS11b and AKAP1 on extended 3’UTR of 3.5-kb mRNA. 3.5-kb mRNA control translation and mRNA turnover at the mitochondria. There is probable differential targeting of MAM complexes by 1.6-kb form, which predominates later in MA10 cells. **(B)** SIK/CRTC control. PKA-mediated induction of STARD1 occurs via reversal of basal SIK/CRTC suppression. Two forms of SIK and CRTC are each maintained inactive through binding of phosphorylated forms by 14-3-3. They are activated by PP2 phosphatases. CRTC2 stimulates CREB when dephosphorylated. CRTC3 partners CRTC2 by enhancing PP2A phosphatase. Suppression of CRTC forms is delivered by basal SIK2 and inducible SIK1, which are, respectively, cytoplasmic and nuclear in their active dephosphorylated forms. **(C)** Pulsatile Control. An increase and decrease of cAMP and PKA over 20–30 min are produced by the normal pulse delivery of ACTH, in combination with cell PDE forms. SIK2 suppression mirrors this time course. SIK1 is induced in an inactive S577 phosphorylated form by high cAMP in the early phase, which then suppresses late in the pulse as cAMP falls. The delay in splicing and late SIK1 inhibition leads to low mRNA that persists between pulses. **(D)** Dual STARD1 activities. STARD1 is active in CHOL import and IMM metabolism at CYP11A1 (11A) when NTD cleaved form, p30, relocates to OMM. The additional activities of p30 STARD1 in IMS, IMM and matrix are poorly defined, particularly activities at CYP27A1 that lead to LXR activation.

In MA10 cells, there is minimal detectable basal STARD1 mRNA and p-RNA, but after Br-cAMP stimulation, loci become asymmetrically activated over the course of 60 min (149) (**Figure 5C**). p-RNA appears at single loci within 15 min, prior to detectable spliced, sp-RNA. After 60 min, which corresponds to the PCR steady state, most cells contain nuclei that express two loci with p-RNA and substantial sp-RNA. Although the RNA at loci is detectable with one transcript, at 60 min levels are in the range of 5-30 transcripts. mRNA is resolved as single particles that requires higher sensitivity and is only detectable after 60–75 min (**Figure 5C**). p-RNA declines between 90 and 180 min, leaving sp-RNA to predominate. mRNA starts to spread from the nuclear envelope after 90 min (**Figure 5E**, 90 min). At 180 min, there is appreciably less sp-RNA at loci, as cytoplasmic mRNA is abundant. In this phase, extra loci become activated in these tetraploid cells. We are evidently viewing a transition to the rapid splicing of coupled transcription (**Figure 7A**).

### Coupling of STARD1 Translation, NTD Import, and Cholesterol Metabolism in Y-1 Cells

**Figure 6:**
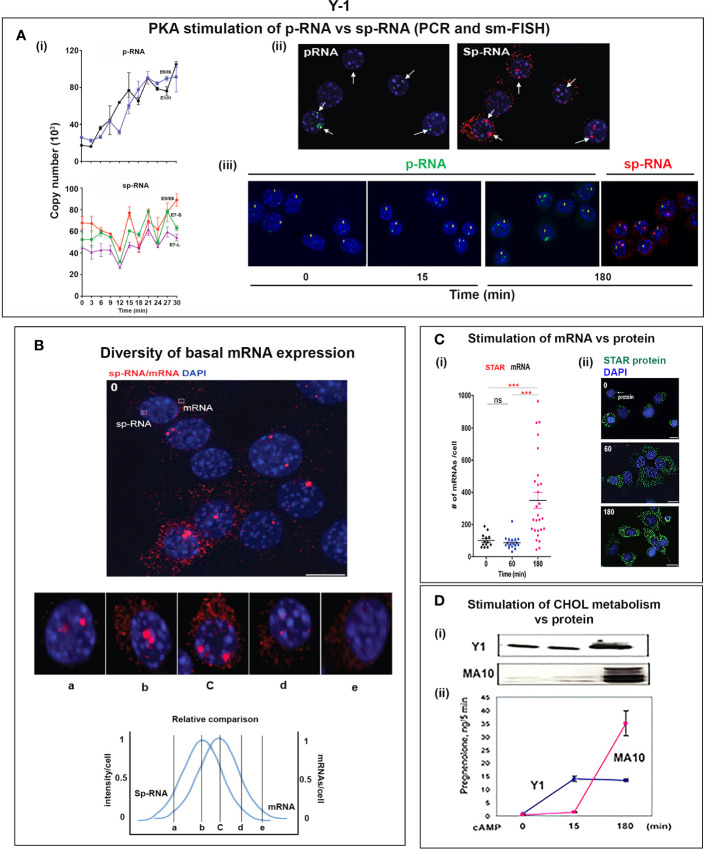
Y-1 cells show heterogeneous basal activation and acute, sustained loci activation with slow splicing and protein translation. **(A)** (i). Time course (qPCR copy number) for stimulation of STARD1 p-RNA (intron1 vs. intron6 (UPPER) and spRNA/mRNA (exon5/exon6; 3’UTR early (S) and extended (L) (LOWER). (ii). sm-FISH basal expression of same 5 cells probed with p-RNA and sp-RNA. The data demonstrate little relationship between p-RNA or sp-RNA at loci and mRNA in cytoplasm. (iii). p-RNA increases between 0 and 15 min but remain similar at 180 min. sp-RNA/mRNA at 180 min increase in a number of cells, with high expression in nucleus and cytoplasm. **(B)** Basal sp-RNA heterogeneity at high sensitivity captures both loci and mRNA. Varying proportions of locus activation and mRNA (a, b, c, d) may reflect different stages of a cycle comprising locus activation, mRNA processing, export, locus inactivation, mRNA turnover. **(C)** (i). Cytoplasmic mRNA particles counted from each of 30 adjacent cells (particle counts/cell) compared to protein expression (cell fluorescence): half the cells do not express detectable cytoplasmic mRNA. Delayed increase in cytoplasmic mRNA occurs from 60-180 min. ***p<.001; ns, not significantly different. (ii). Proportion of cells with high translated protein in mitochondria increases progressively 0–180 min. **(D)** (i). Total protein measured by immunoblot only increases after 15 min in Y-1 cells. (ii). Activity is fully increased in the first 15 min. MA10 activity increases only after 15 min and in parallel with increased protein accumulation. Portions of this figure were previously published (10, 145, 150).

Y-1 cells show appreciable basal p-RNA and sp-RNA in q-PCR analyses. Following stimulation by Br-cAMP, p-RNA rises to a steady-state after 30 min that matches 60-min levels in MA10 cells (**Figures 5A**, **6Ai****)**. The basal sp-RNA, which includes mRNA, is appreciable and does not increase over the initial 30 min (150). This expression approximates the level in MA10 cells after 60 min of stimulation (**Figure 5A**). Sm FISH analysis of Y-1 cells shows basal p-RNA in most cells, but typically only one transcript in a single locus (**Figure 6Aii****)**. The 15-min stimulation, measured by PCR, corresponds to increased p-RNA at these single loci. sp-RNA levels at the loci increases in parallel with the p-RNA (**Figure 6Ai****)**. Thus, the levels of p-RNA and sp-RNA in Y-1 loci at 15 min look very similar at 180 min (**Figure 6Aiii****)**.

Examination of the Y-1 cell basal expression at higher sensitivity shows that about 20% of the cells have extensive cytoplasmic mRNA (**Figure 6B**). In addition, the proportion of sp-RNA at loci and mRNA in the cytoplasm varies extensively. Cytoplasmic mRNA can be assessed by counting the cytoplasmic particles in each cell. Examination of a field of about 30 adjacent cells captures the variability of expression (approximately 20-200) (**Figure 6Ci****)**. Stimulation by Br-cAMP for 60 min does not increase this count, whereas there is a substantial increase at 180 min with extensive difference between individual cells (20–1,000). Protein expression seems to be uncoupled from mRNA. Expression increases after 60 min and still further after 180 min. (**Figure 6Cii****)**. Western blots show that protein increases after 15 min (**Figure 6Di****)**. In this 15-min period, maximum CHOL metabolism is attained (**Figure 6Dii****)**; coupled activity cycle]. For MA10 cells, the resistance to CHX (**Figure 3B**) indicates a different limiting step.

### Exceptional Uncoupled Transcription of STARD1

PKA activates the 300 bp STARD1 proximal promoter through four interacting sites that mediate signaling from CREB, SF1, GATA4 and AP1 (5, 151–153). Each of these four activities may be essential, but PKA stimulation of CREB activity through changes in SIK/CRTC is required for STARD1 stimulation on the 30-min time scale of the hormonal stimulatory pulses (150, 154).

In Y-1 and MA10 cells, STARD1 expression is characterized by the striking retention of a high proportion of unspliced primary transcripts at the loci. We have previously shown that this primary pool is spatially separated at the locus from the spliced pool. Elongation occurs through all six introns prior to initiation of splicing. This spatial separation may occur if primary transcripts accumulate at an intermediate position in elongation due to a pause in Pol2 activity; for example, at the early part of the 3’UTR, which corresponds to the early part of exon 7 and the end of the translated sequence. This possibility is supported by an apparent delay in elongation through the 3’UTR (**Figure 5A**). In MA10 cells, there is a transition to coupled transcription after 60 min, characterized by the increase in sp-RNA at the loci without a change in p-RNA (**Figure 5A**). In MA10 cells, p-RNA at loci disappears by 180 min and sp-RNA also declines, suggesting an increased rate of processing, notably polyadenylation in the 3’UTR. The activation of third and fourth loci in these tetraploid cells suggests a marked change in the cells in this 2- to 3-h time period. These is no decline in coupling in Y-1 cells, which remains at levels similar to those in MA10 cells prior to this transition. This transition in MA10 cells matches a decline in the ratio of 3.5-kb mRNA relative to the less regulated 1.6 kb-form. It is also notable that the onset of STARD1 association with multi-protein aggregates and HSP78 initiates in this period. These features of the coupling of elongation and splicing at the STARD1 loci are summarized in **Figure 7A**.

Atypical features of STARD1 transcription include early Pol2 pausing (basal Y-1 cells), slow splicing and alternative 3’UTR polyadenylation (MA10 and Y-1 cells) (155–158). The 15–20 min delay in splicing is much longer than the time for primary elongation through intron 6 (**Figure 5A**; <2 min). The delay may derive from slow PKA induction of a limiting factor. There is also a progressive, slow association of CRTC2 with the splicing factor, SC35 (150, 159). ACTH stimulation of the expression of splicing factors in Y-1 cells has also been described (160).

### SIK/CRTC Activation Mediates PKA Control of STARD1

Recruitment of CBP to ^p133S-^CREB is enhanced by partnership between CRTC2 and CRTC3, which are each maintained in an inactive state in the cytoplasm through phosphorylation by SIK2 and sequestration by 14-3-3 proteins (111, 143, 161). PKA rapidly phosphorylates and inactivates SIK 2 (S562), and the phosphorylated CRTC forms (CRTC2: S171, 275) are then activated by cytoplasmic PP2 phosphatases, notably Ca^2+^-activated PP2B (calcineurin). This cascade effects CRTC transfer to the nucleus and association with ^p133S-^CREB (162, 163). SIK1 functions as an additional nuclear suppressor by restoring phosphorylation to CRTC2. SIK1 has a nuclear localization sequence (absent in SIK2), and dephosphorylation by PP2A effects transfer to the nucleus. SIK1 inhibition is opposed by nuclear CRTC3, when phosphorylated at S391 by MEK-Erk kinases. CRTC3 then enhances recruitment of the PP2A phosphatase to CRTC2 (162) (**Figure 7B**).

The scheme in **Figure 7C** speculates how this SIK CRTC signaling fits within a 30-min pulse of ACTH or even LH. STARD1 pRNA selectively increases at loci after 15 min, which corresponds to about 75% of the pulse stimulation, but with very little mRNA. This disparity has been demonstrated *in vivo* (30, 136). ACTH is distinguished from Br-cAMP by a substantial contribution from Ca^2+^ and Ca kinases. cAMP potently dissociates the diverse repressive regulatory subunits of PKA forms 4 and 8. The removal of cAMP by PDE forms is critical in precisely attenuating PKA activity during the pulse. cGMP also plays a role in *in vivo* steroidogenesis, notably in the testis (142). This is made evident by the participation of the cGMP-specific form, PDE5.

**Figure 8:**
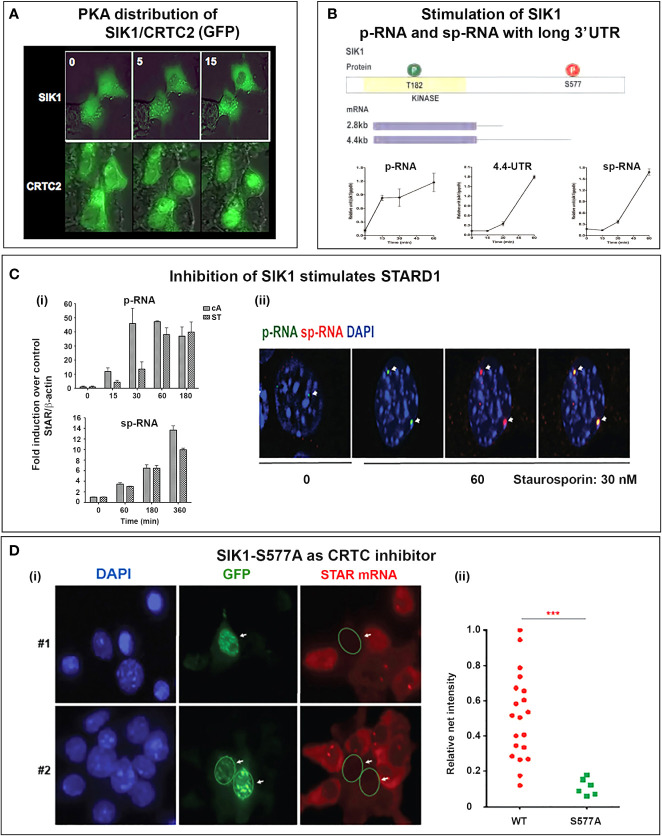
Activation of SIK1 and CRTC2; inhibition by SIK1S577A GFP. **(A)** SIK/CRTC responses in Y-1 cells. Inverse nuclear transfer of SIK1-GFP and CRTC2-GFP is stimulated with Br-cAMP. **(B)** Stimulation of SIK1 pRNA and 10.7554/eLife.25466 mRNA (total and extended 4.4-kb forms). **(C)** Stimulation of STARD1 by inhibition of SIK forms. (i). Inhibition with 30 nM staurosporine (ST) compared to Br-cAMP (cA, 0.4 uM) (p-RNA/pre-mRNA and sp-RNA/mRNA). (ii). sm-FISH images of STARD1 p-RNA and sp-RNA at loci after 180 min with 30 nM ST. **(D)** Inhibition of CRTC by transfection with SIK1-S577A-GFP. (i). Inhibition of STARD1 mRNA in Y-1 cells, but not in adjacent non-transfected cells. (ii). Expression comparison of STARD1 mRNA particles in 26 adjacent cells from the same culture, with (S577A) and without (WT) transfection. Portions of this figure were previously published (145, 150, 164). ***p<0.001.

The delayed appearance of SIK1 during the ACTH decline phase may be expected to attenuate p-STARD1 during the latter part of the pulse when ACTH and cAMP are declining. Importantly, we know little of the kinetics of inactivation of CRTC2 as PKA declines. This may be expected to depend on the interplay between CRTC3, PP2 and SIK1 (**Figure 7B**). This model would result in STARD1 mRNA levels that depend on the pulse frequency. SIK1 has low basal expression of p-RNA and sp-RNA, but, like STARD1, responds rapidly to PKA, as both a primary transcript and predominant extended 3’UTR transcript (4.4-kb form) (**Figure 8B**). Typical of a labile mRNA, steady state expression is attained in 60 min, with a large increase within 20 min. This delay corresponds to the period of the hormonal cAMP pulses.

STARD1 is involved in the activation of CHOL metabolism at CYP27A1 and LXR. The role of the NTD in STARD1-dependent processes varies according to tissue and stimulation. The processing of STARD1 also appears to be slower in testis cells than in adrenal cells. Pulsatile activations vary, notably according to the diurnal timing. The contributions of the MAM mechanisms, including TSPO complexes, are likely to vary, notably with diet. In **Figure 7D**, we provide a scheme that includes the possibility that imported p30 can refold like other imported proteins and contribute to the CHOL access to CYP27A1. We have previously seen that inhibition of CYP11A1 with AMG causes an increase and subsequent decrease of reactive IMM CHOL over the course of 4 h (60). A matrix START presence may contribute to such protection.

The SIK1 and CRTC2 proteins can be tracked in real time, when fused with GFP. **Figure 8A** shows time-lapse images following Br-cAMP stimulation of Y-1 cells. Under basal conditions, although both are present in nuclei and cytoplasm, SIK1 is more nuclear and CRTC2 more cytoplasmic. Within 5 min, this selectivity reverses, and after 15 min, phosphorylated SIK1 is fully cytoplasmic and CRTC2 is almost fully nuclear. During this period STARD1 transcription is increasing (**Figure 6A**).

The importance of SIK forms for PKA induction of STARD1 is demonstrated by the finding that chemical inhibition of SIK forms, with very low concentrations of staurosporine (ST) (30nM), elevates STARD1 pRNA and sp-RNA/mRNA to levels reached with Br-cAMP. p-RNA responds approximately three-fold slower to ST than to PKA (**Figure 8Ci****)**. There is also a delay for stimulation of sp-RNA by ST, which activates CRTC2 and STARD1 with minimal phosphorylation of CREB. Thus, while CREB phosphorylation enhances CRTC2 and CBP binding, phosphorylation is not required for effective transcription. Like PKA, ST activates both p-RNA and sp-RNA to similar levels in the loci, with no spatial separation (**Figure 8Cii****)**. ST activation does not occur in MA10 cells, where the broad specificity kinase inhibitor also attenuates PKA activation. A more selective, but less potent SIK inhibitor (HG 9-91-01) (165) produces equivalent stimulation of STARD1 in MA10 cells.

SIK1 remains inactive in the cytoplasm when phosphorylated by PKA (S577). However, the S577A mutation maintains this form in nuclear speckles that co-localize with CRTC2. The GFP fusion with S577A SIK fully inhibits CRTC processes in all transfected cells marked by GFP (**Figure 8Di****)**. We established full suppression of STARD1 mRNA compared to neighboring non-transfected cells (**Figure 8Dii****)** (150). As noted earlier, SIK1 also functions in the cytoplasm when activated by CAMK1, including for activation of SR-B1 (111).

SIK forms also require activation by the kinase, LKB1 (154, 166). AMPK shares this activation by LKB1 and can replicate much of the CRTC inhibition delivered by SIK, although with activation delivered by metabolic deprivation of ATP. As noted earlier, AMPK also suppresses mTORC1 kinase activty (**Figure 4**). This suppression extends to SIK forms.

### Dual 3’UTR STARD1 Transcripts; Selective Regulation of the Extended 3’UTR by TIS11b and AKAP1

**Figure 9:**
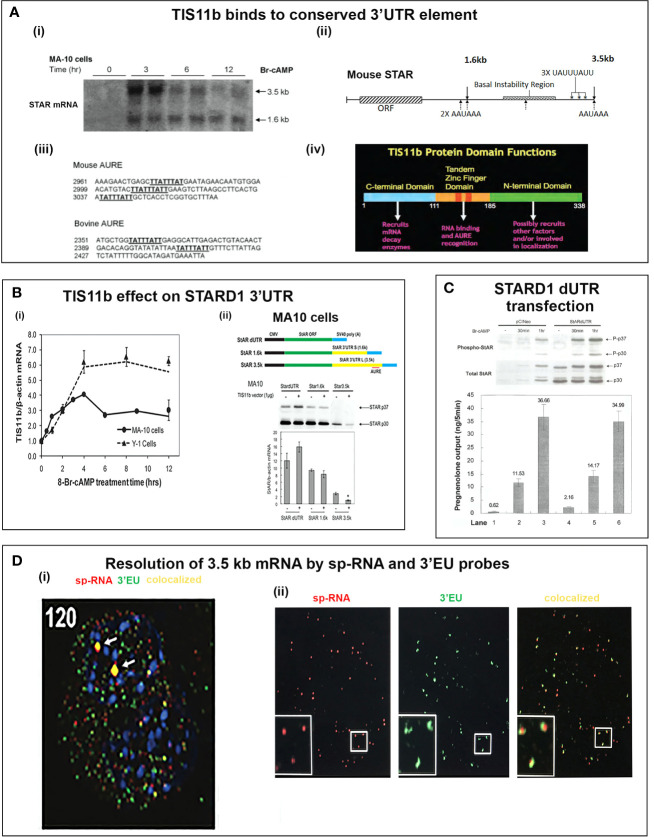
Expression of STARD1 3.5- and 1.6-kb mRNA. Selective targeting of TIS11b to 3.5 kb form. **(A)**. (i). In MA10 cells, Br-cAMP stimulates the 3.5-kb form for 3 h, followed by a decline. The 1.6-kb form is constant. (ii). Diagram of STARD1 3’UTR sites on 1.6 and 3.5-kb mRNA. (iii). The extended 3’UTR retains two or three 8-base TIS11b binding sites in mouse and bovine sequences. (iv). Domain structure of TIS11b. **(B)** (i). Y-1 and MA10 cells both show Br-cAMP-mediated increases in TIS11b expression. (ii). Co-expression of STARD1 forms with different 3’UTR. TIS11b selectively suppresses 3.5-kb form and STARD1 protein. **(C)** In MA10 cells, impact of supplemental transfection of dUTR STARD1 on S195 phosphorylation, NTD cleavage and pregnenolone formation. Early CHOL metabolism is increased, but not 30- and 60-min stimulations. The p37/p30 ratio is much higher for phospho-STARD1. **(D)** (i). sm-FISH analysis in Y-1 cells shows overlap (yellow) of ORF/sp-RNA (red) and extended 3’UTR/3’EU (green) at the loci after stimulation for 120 min. (ii). sp-RNA/ORF and 3’UTR/3’EU probes each bind 3.5-kb mRNA. Seventy percent (see inserts) of mRNA show 3’EU (green), half paired with sp-RNA/ORF (red). Thirty percent only bind sp-RNA/ORF (red). Portions of this figure were previously published (150, 164).

STARD1 is transcribed as two alternatively polyadenylated mRNA, with short (1.6 kb) and long (3.5 kb) 3’UTR. Northern blots of MA10 and Y-1 mRNA show both 3.5- and 1.6-kb forms, in an approximate 3:1 ratio over the initial 180-min post stimulation period. In Y-1 cells, total sp-RNA is maintained for 12 h (167). In MA10 cells, there is a selective loss of 3.5-kb mRNA between 3- and 6 h, thus leaving the 1.6-kb form predominating after 12 h (**Figure 9Ai****)**. These secondary changes in MA10 cells occur in parallel to the onset of the GRP78-mediated appearance of STARD1 at MAM sites (**Figure 3D**). The diversity of single cell STARD1 expression is very striking and is likely to be reflected in their CHOL metabolism.

The 3’UTR of the 3.5-kb mouse STARD1 has equivalent extended overall in bovine and human forms, but with minimal overall conservation, except for sets of 2-3 UAUU*UAUU* elements in the terminal regions (168, 169) (**Figures 9Aii, iii****)**. These elements bind dimers of the zinc finger protein, Znf36l1/TIS11b (**Figure 9Aiv****)**, which is stimulated by PKA (**Figure 9Bi****)**, in parallel with STARD1. TIS11b selectively enhances turnover of the 3.5-kb mRNA, with no effect on the 1.6-kb form, which lacks the binding elements (**Figure 9Bii****)** (168–170). TIS11b also forms specific RNA particles (171).

AKAP1, which is essential for steroidogenesis, attaches to the OMM and binds to KH domains adjacent to TIS11b sites (144). This association fixes the mitochondrial location of 3.5-kb mRNA, while also sequestering the PKA that phosphorylates the emerging p37 protein (65, 144, 172). AKAP1 also directs the DRP1 GTPase activity that completes the mitochondrial fusion-fission cycle (173), a potentially important component of STARD1 activity (37).

STARD1, modified to remove the 3’UTR (STARD1-dUTR) to complex TIS11b or AKAP1, produced the same NTD cleavage products and S195 phosphorylation. Although there was an increase in basal activity, there was no effect after 30 min of Br-cAMP stimulation, despite a more than ten-fold increase in expressed protein (**Figure 9C**). Pulse chase experiments with normal and PKA-deficient Y-1 cells show that phosphorylation does not affect the matrix turnover of STARD1 (52). Import occurs without PKA activation and yet there is no CHOL metabolism (56). p37 NTD-STARD1 is shown here to be more heavily phosphorylated by PKA than the cleaved matrix forms. There is evidently an effect of S195 phosphorylation on the balance of formation, import and cytoplasmic degradation. The import is 40 times faster than matrix turnover by matrix proteases, notably Lon (67).

We used sm-FISH to localize the mRNA in the cell. About 25% of the mRNA only hybridize with sp-RNA, consistent with the proportion of the 1.6-kb mRNA. The remainder hybridize the 3’-EU, consistent with the proportion of 3.5-kb mRNA (**Figures 9Di, ii****)**. Half of the mRNA bound by 3’-EU also hybridized the sp-RNA probe, but half failed to bind sp-RNA probes. These mRNA may be protected by engagement in translation of mRNA at the mitochondria.

### sm-FISH Resolution of STARD1 mRNA Interaction With Mitochondria

**Figure 10:**
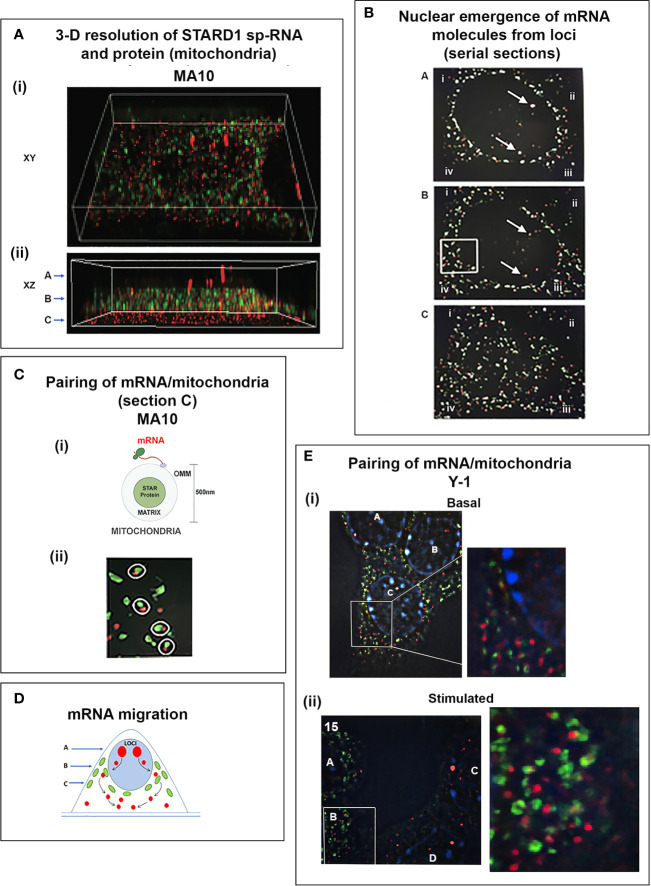
sm-FISH identification of single cell location of STARD1 mRNA in relation to gene loci and mitochondria. Identification of 1:1 mRNA-mitochondria association. **(A)** MA10 cell 3D assembly of STARD1 sp-RNA/mRNA and protein/mitochondria 180 min after Br-cAMP stimulation. (i). XY shows top view; (ii). XZ shows side view. **(A–C)** arrows indicate levels used for three optical slices in B. Red: sp-RNA at loci (intense) and in cytosol as mRNA; Green: STARD1 protein in mitochondrial matrix. Studies with Mitotracker and STARD1 protein show near total overlap. **(B)** In slices A and B, mitochondria with high STARD1 protein (green/white) mark the nuclear envelope, which they encircle. Same two nuclei (red/white; arrows) are detected in each section (slice A>slice B). Single mRNA particles (red) move from loci to nuclear pores. Outside the nucleus, many mRNA pair with single mitochondria marked by STARD1 protein (boxed area enlarged in section C). Slice C is below the nucleus. mRNA less frequently paired with mitochondria (see section A). **(C)** (i). Diagram shows spacing for mRNA attached to ribosome, separated from STARD1 protein in the mitochondrion matrix. (iii). Pairing of mRNA and mitochondria (box slice B). **(D)** Diagram shows movement of mRNA particles from loci to lower cytoplasm zone. **(E)** Y-1 cells imaged for STARD1 sp-RNA/mRNA and protein/mitochondria. (i). 3 basal cells **(A, B, C)** marked by DAP1 nuclei each show STARD1 mRNA (red) and protein (green). (Box Enlarged) Cell C; 15-20 mRNA particles and mitochondria/STARD1 with six examples of mRNA/mitochondrion pairs. (ii). 15-min Br-cAMP stimulation (four cells). Similar pairing of mRNA and mitochondria. Some mitochondria cluster without mRNA. Portions of this figure were previously published (146, 150).

In MA10 cells, STARD1 mRNA transfers the protein into all mitochondria identified by Mitotracker after a 180-min Br-cAMP stimulation (145). Overlap of mRNA and protein is far less complete. One such cell has been assembled from a series of XY slices into a 3D reconstruction (**Figure 10Ai****)**. The cell resolves into distinct functional zones (**Figure 10Aii****)**. Y-1 cells show similar features prior to and following 15-min Br-cAMP activation (**Figures 10Ei, ii****)**.

Loci in both cell types are positioned high in the nucleus (**Figure 10B**). In these MA10 cells, two loci, within the nucleus, are ringed by mitochondria that express STARD1 protein. At higher sensitivity, single mRNA particles are imaged tracking between the loci and the peri-nuclear mitochondria (**Figures 10Ci, ii****)**. Although this movement of mRNA nucleoprotein particles is reported to occur through diffusion, the directionality is clear (174) (**Figure 10D**). In a central plane, over half of the mRNA pairs with single mitochondria (pair diameter within 1 um) as they emerge from nuclear pores. In the next plane, at the base of the nucleus, mRNA particles have separated from mitochondria. There is an apparent movement of mRNA downward from the loci, through a zone of mitochondria where association is followed by a zone close to the PM, in which mRNA accumulate fully separated from mitochondria. This trafficking is fully consistent with dynein-mediated microtubule movement of mRNA. We envisage 3.5-kb mRNA engaged in translation coupled CHOL transfer within the paired structures. Electron cryotomography studies of yeast mitochondria show that relatively few mRNA molecules bind to a mitochondrion at a single time. However, arrest of translation with CHX increases their presence (175).

This mitochondrial-mRNA pairing is also evident in Y-1 cells under basal conditions or after 15 min of stimulation that produces peak CHOL metabolism (**Figure 10E**). These 1:1 associations appear in a similar proportion to the mRNA, but not to the sp-RNA probe. This selectivity is consistent with the model of mitochondrial mRNA pairing shown in **Figure 10D**. Some insert images also capture mitochondrial clustering in absence STARD1 mRNA, perhaps after DRP-mediated fission.

## SM-Fish Analysis STARD1 after Stimulation by HCG In Adult Mouse Leydig Cells Compared to MA10 Cells

Testis Leydig cells depend on their microenvironment within the architecture of the whole testis and, particularly, on their crosstalk with Sertoli cells and on local CHOL transfer processes. Mammalian testis has two functional compartments, seminiferous tubules and the interstitium between the tubules, each with interdependent roles. Sertoli cells surround germ cells within the tubules and support spermatogenesis (176). Sertoli cells also have a major role in the intra-testis delivery of CHOL. Leydig cells, located in the interstitium, are specialized cells expressing steroidogenic genes for testosterone synthesis (177), but also express genes that convert CHOL to bile acids that repress testosterone synthesis (26, 27). Neonatal depletion of Sertoli cells decreases the numbers of adult Leydig cells in mice (178), indicating regulatory crosstalk between these cell types. The secretion of testosterone by adult Leydig cells is primarily under the control of pituitary-derived LH. After the onset of puberty, Leydig cells are stimulated by regular 15-min pulses of LH at roughly an hourly frequency when it stimulates the expression of steroidogenic genes, including STARD1, Cyp11a1 and Hsd3b2, among other activities (179–181).

**Figure 11:**
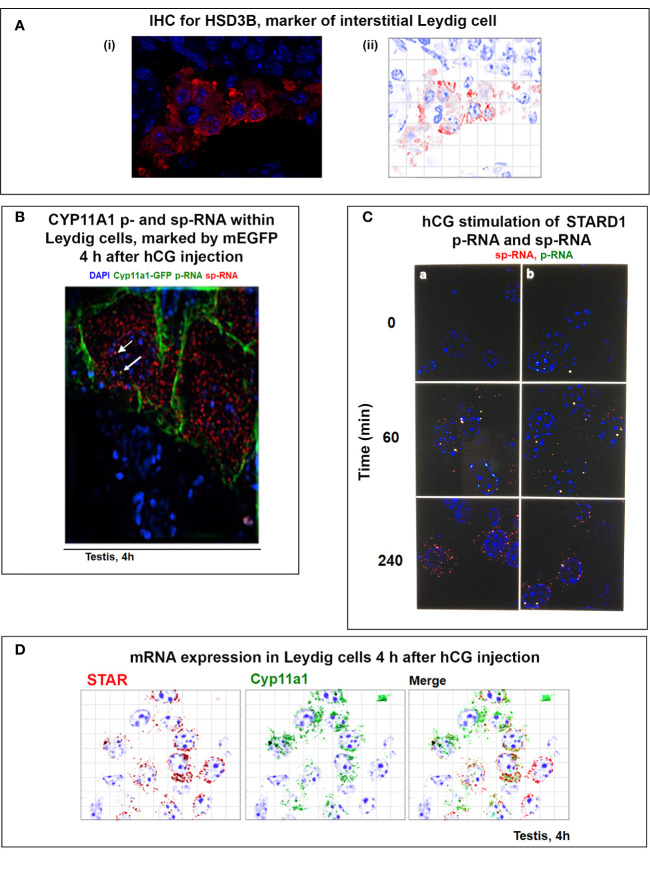
sm-FISH facilitates visualization of STARD1 and Cyp11a1 primary and spliced transcripts at the resolution of single Leydig cells after hCG injection of adult male mice. **(A)** Immunohistochemistry of 3b-HSD protein (unaffected by hCG). Left: Standard 100x image of a single interstitial zone; Right: 3D deconvolution. **(B)** Two Leydig cells after 240-min stimulation of STARD1 mRNA (red) resolved by NSIM. Cells are bordered by EGFP that locates to the plasma membrane of Leydig Cells in Cyp11a1Cre+;mTmG mice. A single locus is expressing p-RNA (green in nucleus). **(C)** sm-FISH analysis of STARD1 loci for duplicate sections (p-RNA, green; sp-RNA, red; overlap, yellow) at 0-, 60-, and 240-min post hCG stimulation. 0 min: 1 p-RNA/9 cells; 60 min: 10/11 cells with loci (many with dual p-RNA/sp-RNA presence-yellow); 240-min variable loci express RNA in parallel with mRNA (low sensitivity). Columns a and b represent different populations of cells within the same section of the testis. **(D)** Images of interstitial cells following 240-min hCG stimulation, 3D de-convolution for Cyp11a1 and STARD1 (high sensitivity). Cyp11a1 shows similar expression at basal and induced conditions (not shown). STARD1 shows similar induced mRNA levels but with different positioning. Portions of this figure were previously published (182).

A standard method to detect Leydig cells is the use of immunohistochemistry with a steroidogenic enzyme marker, such as HSD3B (**Figure 11A**). To facilitate visualization of primary and spliced transcripts specific to Leydig cells, *in vivo*, genetic mouse models, *Cyp11a1-Cre^+^ and mTmG*, were crossed to target expression of EGFP within the cell membrane of Leydig cells (183, 184). Adult male *Cyp11a1-Cre^+^, mTmG* mice were injected with hCG and testes were harvested at 0, 1, and 4 h to evaluate transcript expression specific to Leydig cells. hCG stimulates Leydig cells through the LH receptor, as LH, but is resistant to the rapid proteolytic turnover that affects the natural activator. **Figure 11B** shows the high level of mRNA (sp-RNA, red stain) that is expressed 4 h after administration of hCG. The sm-FISH image shows resolution of primary transcripts within the DAPI-stained nucleus (p-RNA, single green dot at locus, arrow) and spliced transcripts (sp-RNA, red) as particles within the nucleus and cytoplasm of labeled Leydig cells highlighted by EGFP-labeled cell membrane (green). The density of mRNA particles probed by sp-RNA parallels that seen in MA10 cells (**Figure 7C**). A second locus is marked in this cell by sp-RNA, corresponding to asymmetric locus activation, a feature for MA10 cells (**Figure 11B**).

Images of 8–10 stimulated Leydig cells show a progressive effect of hCG on STARD1 expression (**Figure 11C**). Only a single unspliced locus is apparent under basal conditions (time 0, panel b). After 60 min, half of the cells have loci with dual labeling for p- and sp-RNA, typical of slow splicing, while a few exhibit only spliced loci. Thus, loci respond in an asymmetric manner. After 240 min, there is a decline in expression at loci within the nucleus, but an increase in mRNA within cytoplasm. The trend parallels that seen in MA10 cells after Br-cAMP stimulation (**Figure 7**). Although CYP11A1 and STARD1 proteins each locate to mitochondria, their mRNA show very different cell distributions (**Figure 11D****)**. These differences may arise because STARD1 introduces extra elements of positional selectivity, with the novel targeting provided by the NTD and the extended 3’UTR of the 3.5-kb mRNA.

## Conclusions

These *in vivo* testis experiments on STARD1 expression establish robust parallels with the expression mechanisms in MA10 cells (**Figures 7**, **11**). Several features are shared with Y-1 cells; slow splicing and mRNA processing and some selective, low basal expression **(****Figures 8**, **9****)** (**Table 1**). The shared characteristics of the synthesis of STARD1 mRNA, in part, reflect the involvement of the SIK/CRTC CREB pathway that is well adapted to pulsatile stimulations delivered by ACTH and LH to their respective receptors (**Figure 5**). This slow splicing is also seen in the ACTH pulse stimulation of the adrenal gland, *in vivo*, where primary transcripts also predominate. The same is reported, here, for hCG stimulation of Leydig cells in the testis. We see this in the loci of individual cells where, again, the marked asymmetry of stimulation is apparent. This pulsatile signaling is also compatible with the rapid cycling identified in the ACTH stimulation of rat adrenals, *in vivo*, and Y-1 cells, *in vitro*. Although MA10 cells differ with respect to stronger basal suppression, the distribution of RNA species in MA10 after 60 min of stimulation is very similar to that in Y-1 cells, even with 15 min of stimulation when CHOL metabolism is maximum.

**Table 1:** Cell type dependence of STARD1 mechanism.

Characteristic	Y-1 Adrenal	MA10 Testis	*In vivo* Testis
Early Uncoupled Splicing	Yes	Yes	Yes
Sustained slow splicing	Yes	**NO**	Yes
Delayed mRNA increase	YES	YES	YES
3.5kb mRNA dominant	YES	YES	YES
Basal pRNA, spRNA, mRNA	YES	NO	**NO**
Asymmetric Locus Activation	YES	YES	YES
Paired mRNA-Mitochondria	YES	YES	?
Deletion activated LD	YES	YES	YES
CRTC2 SIK S577A inhibit	YES	YES	?
Stimulation by SIK Inhibitor	YES	YES	?
High SIK1 induction	YES	YES	?
Acute Translation-Coupled CHOL Metabolism	YES	?	?
Delayed ER-MAM Complex	?	YES	?

See **Figure 5** for graphic representation; **Figures 7****–****11** for data.

sm-FISH microscopy shows the activation of STARD1 loci and mRNA in adrenal and testis cells, at the level of single RNA molecules in individual cells (**Figures 10A, B**, **11C**). A single p-RNA transcript holds many basal Y-1 loci in a pro-active status, even as neighboring cells are engaged. Both cells maintain near equal levels of p-RNA and sp-RNA, indicative of similarly slow splicing. Single mRNA are visible, tracking between loci and nuclear membrane pores, and then interacting 1:1 with single mitochondria that express STARD1 protein. In a lower section, most mRNA particles and mitochondria have separated. In the 3D reconstruction of stimulated MA10 cells, a route for passage of mRNA from the upper loci to a terminus is suggested by a band of mRNA near the adherent PM. This route may represent a microtubule/dynein track. **Figure 9A** shows a 3.5- and 1.6-kb mRNA at a ratio of 3:1. In **Figure 9C**, consistent with this ratio, 70% of mRNA molecules bind 3’EU that marks the 3.5-kb form, while 30% bind sp-RNA that marks the 1.6 kb-form. Half of the 3.5-kb mRNA bind the 3’EU probes, but not the sp-RNA probes that cover the ORF. Critically, we need to establish a presence of this 3.5-kb form in the 1:1 complex that probably captures the translation-coupled mitochondrial import of CHOL and StARD1 (**Figure 3A**).

StARD1 does not need the NTD to activate CHOL transfer across the OMM/IMM barrier in adrenal cells, but the NTD is necessary for Leydig cells. Interestingly, TSPO is needed for the adrenal process much more than in the testis. The NTD evidently serves a purpose, first as a key part of the translation coupled CHOL import process. The tissue differences may reflect differences in mechanism: Leydig cells integrating with Sertoli cells in the testis; greater demands on STARD1 for CYP27A1 and the LXRα pathway to ABCA1 and nHDL (**Figure 3B**).

A high energy price is paid to generate the 3.5-kb form and yet this is largely ignored. Mice and humans generate this extend 3’UTR, but with a minimum of shared sequence, except for conserved repeats of the TIS11b recognition sequence. However, TIS11b destabilizes STARD1 3.5-kb mRNA and yet increases CHOL metabolism. We show that the 3.5-kb mRNA is remarkably potent at sustaining mitochondrial CHOL metabolism, in large part through 3’UTR interactions with AKAP1 and TIS11b. Removal of the 3’UTR maintains full phosphorylation and import of CHOL but fails to enhance endogenous CHOL metabolism.

The extra precision introduced by translation-coupling, intervention of labile pRNA, 3.5-kb mRNA instability and SIK/CRTC regulation each introduces speed and flexibility that fit both pulsatile stimulation and the changing demands of mitochondrial fusion/fission cycles. The countering involvement of a slow acting MAM/ER mechanism remains to be addressed in adrenal cells.

STARD1 does not need the NTD to activate CHOL transfer across the OMM/IMM barrier in adrenal cells, but the NTD is necessary for Leydig cells. Interestingly, TSPO is needed for the adrenal process much more than in the testis. The NTD evidently serves a purpose, first as a key part of the translation coupled CHOL import process. The tissue differences may reflect differences in mechanism between adrenal and testis. Differences in the participation of NTD forms (**Figures 3Ci, ii****)** and TSPO may arise from changes in the physiological demands including pulsatile stimulation (**Figure 7C**), high rates rate of CHOL metabolism during adrenal stress or participation of alternative mechanisms, including the CYP27A1/LXR regulation of CHOL trafficking (**Figure 7D**) and the protection against CHOL-induced stress.

## Author Contributions

CJ completed the writing and conceptual design. ML continued refinement of protocols and completed sm-FISH and qPCR experiments and assisted in editing and figure preparation. JL established protocols and completed sm-FISH and qPCR experiments. JSJ led the testosterone contribution in the in vivo testis. All authors contributed to the article and approved the submitted version.

## Funding

This work was supported by the National Institutes of Health [grant number: R01 HD090660].

## Conflict of Interest

The authors declare that the research was conducted in the absence of any commercial or financial relationships that could be construed as a potential conflict of interest.

## Abbreviations

ABCA1, ATP-binding cassette subfamily A member 1; ACAT1, acetyl-CoA acetyltransferase; AKAP1, A-kinase anchor protein 1, mitochondrial; ApoA1, apolipoprotein A1; CBD, cholesterol binding domain; CHOL, cholesterol; CHX, cycloheximide; CREB, cAMP response element-binding protein; CRTC, CREB regulated transcription coactivator; DRP1, dynamin related protein 1; EE, early endosomes 3’UTR, 3’Untranslated region of mRNA; 3’EU, 3’UTR in 3.5-kb mRNA but not 1.6kb mRNA; 3bHSD, 3-beta-hydroxysteroid dehydrogenase; hCG, human chorionic gonadotropin hormone; HDL, high density lipoprotein; IMM, inner mitochondrial membrane; LD, lipid droplets; LDL, low density lipoprotein; LE, late endosomes; LH, luteinizing hormone; LXRα, liver X receptor alpha LY, lysosome; MA10 cells, mouse Leydig tumor cell line; MCS, membrane contact sites; mTORC1, mammalian target of rapamycin complex 1; NTD, N-terminal domain; OMM, outer mitochondrial membrane; PDE, phosphodiesterase; PKA, protein kinase A; PM, plasma membrane; p-RNA, primary RNA transcript; PP2A, protein phosphatase 2A; PP2B, protein phosphatase 2B; SC35, splicing factor; SF1, steroidogenic factor 1; SIK, salt-inducible kinase 1; sm-FISH, single molecule fluorescence *in situ* hybridization; sp-RNA, spliced RNA transcript; SR-B1, scavenger receptor, class B type 1; SREBP2, sterol regulatory element-binding protein 2; STARD1, steroidogenic acute regulatory protein; ST, staurosporine; TIS11b, also called ZFP36L1; TSPO, translocator protein, also peripheral benzodiazepine receptor; 3’UTR, 3’ untranslated region; VAP, vesicle associated membrane protein; Y-1 cells, mouse adrenocortical tumor cell line.
